# Comparing influenza vaccine efficacy against mismatched and matched strains: a systematic review and meta-analysis

**DOI:** 10.1186/1741-7015-11-153

**Published:** 2013-06-25

**Authors:** Andrea C Tricco, Ayman Chit, Charlene Soobiah, David Hallett, Genevieve Meier, Maggie H Chen, Mariam Tashkandi, Chris T Bauch, Mark Loeb

**Affiliations:** 1 Li Ka Shing Knowledge Institute of St Michael’s Hospital, Toronto, Ontario, Canada; 2 GlaxoSmithKline, Canada, Mississauga, Ontario, Canada; 3 Faculty of Pharmacy, University of Toronto, Toronto, Ontario, Canada; 4 Institute of Medical Sciences, University of Toronto, Toronto, Ontario, Canada; 5 Applied Health Research Centre, St Michael’s Hospital, Toronto, Ontario, Canada; 6 North America Vaccines Division, GlaxoSmithKline, Philadelphia, PA, USA; 7 Department of Mathematics and Statistics, University of Guelph, Guelph, Ontario, Canada; 8 McMaster University, Hamilton, Ontario, Canada; 9 Faculty of Health Sciences, McMaster University, Michael G DeGroote Centre for Learning, Hamilton, Ontario, Canada

**Keywords:** Antigenic variation, Cross protection, Influenza A virus, Influenza B virus, Meta-analysis, Systematic review, Vaccines

## Abstract

**Background:**

Influenza vaccines are most effective when the antigens in the vaccine match those of circulating strains. However, antigens contained in the vaccines do not always match circulating strains. In the present work we aimed to examine the vaccine efficacy (VE) afforded by influenza vaccines when they are not well matched to circulating strains.

**Methods:**

We identified randomized clinical trials (RCTs) through MEDLINE, EMBASE, the Cochrane Library, and references of included RCTs. RCTs reporting laboratory-confirmed influenza among healthy participants vaccinated with antigens of matching and non-matching influenza strains were included. Two independent reviewers screened citations/full-text articles, abstracted data, and appraised risk of bias. Conflicts were resolved by discussion. A random effects meta-analysis was conducted. VE was calculated using the following formula: (1 - relative risk × 100%).

**Results:**

We included 34 RCTs, providing data on 47 influenza seasons and 94,821 participants. The live-attenuated influenza vaccine (LAIV) showed significant protection against mismatched (six RCTs, VE 54%, 95% confidence interval (CI) 28% to 71%) and matched (seven RCTs, VE 83%, 95% CI 75% to 88%) influenza strains among children aged 6 to 36 months. Differences were observed between the point estimates for mismatched influenza A (five RCTs, VE 75%, 95% CI 41% to 90%) and mismatched influenza B (five RCTs, VE 42%, 95% CI 22% to 56%) estimates among children aged 6 to 36 months. The trivalent inactivated vaccine (TIV) also afforded significant protection against mismatched (nine RCTs, VE 52%, 95% CI 37% to 63%) and matched (eight RCTs, VE 65%, 95% CI 54% to 73%) influenza strains among adults. Numerical differences were observed between the point estimates for mismatched influenza A (five RCTs, VE 64%, 95% CI 23% to 82%) and mismatched influenza B (eight RCTs, VE 52%, 95% CI 19% to 72%) estimates among adults. Statistical heterogeneity was low (I^2^ <50%) across all meta-analyses, except for the LAIV meta-analyses among children (I^2^ = 79%).

**Conclusions:**

The TIV and LAIV vaccines can provide cross protection against non-matching circulating strains. The point estimates for VE were different for matching versus non-matching strains, with overlapping CIs.

## Background

Influenza is a major public health threat. It is widely accepted that an annual influenza vaccination is the most effective way to prevent influenza [1]. Among adults, for example, the inactivated influenza vaccine was reported to have a vaccine efficacy (VE) of 59% (95% confidence interval (CI) 51 to 67) [2].

Recommendations for influenza vaccine composition currently include two type A influenza strains (A-H3N2 and A-H1N1) and one type B influenza strain, which are updated annually. Strains are selected by scientists convened by the World Health Organization (WHO) [3] in the months before the next epidemic season is expected. This procedure includes consideration of antigenic mismatch between vaccine strains and actual epidemic strains.

Although it is accepted that matched strains provide the best protection, data on the protective efficacy of unmatched strains, or cross protection, are sparse. These data are of particular importance, given that influenza B vaccine strains did not match circulating strains in six influenza seasons between 2000 and 2011 in the USA [4]. Mismatched seasons may lead to reduced uptake of influenza vaccination, reduced VE, and more severe influenza epidemics. Therefore, estimating prevention that can be achieved during mismatched influenza seasons is of prime public health importance. This information cannot be gleaned from previous reviews on the efficacy of the influenza vaccines because they did not specifically examine vaccine efficacy for mismatched seasons [5-7]. As such, in the present work we aimed to determine the cross protection against laboratory-confirmed influenza through vaccination.

## Methods

### Protocol

The Preferred Reporting Items for Systematic Reviews and Meta-analyses (PRISMA) statement was used to guide the reporting and conduct of this review [8]. A systematic review protocol was compiled and circulated to experts in influenza, systematic reviews, and statistics. The protocol was registered with PROSPERO, an international registry for systematic reviews (CRD42012001926) and published in an open-access journal [9].

### Eligibility criteria

Studies reporting the incidence of influenza infection after vaccination among healthy individuals were included. The primary outcome was the incidence of laboratory-confirmed influenza verified by polymerase chain reaction (PCR) or viral culture. The secondary outcome was the incidence of laboratory-confirmed influenza verified by at least a fourfold rise in hemagglutinin inhibition (HI) antibody titers at the end of the influenza season versus baseline (serologic assay) or serologic assay in combination with another detection method (for example, viral culture or PCR). Randomized clinical trials (RCTs) and quasi-RCTs (that is, use of non-random methods to allocate patients to the treatment and control groups, such as consecutive enrolment or the last digit of a health card number) comparing any influenza vaccine versus placebo and disseminated in the English language were included.

### Study selection process

The eligibility criteria were pilot tested on a random sample of 50 citations and clarified, as needed. Two reviewers subsequently screened titles and abstracts (citations) from the literature search in duplicate. Conflicts were resolved through team discussion. A similar process was followed for screening potentially relevant full-text articles identified through citation screening.

### Information sources and search

The full search strategy is reported elsewhere [9]. Briefly, RCTs were identified by searching three Cochrane reviews on influenza vaccines [5-7], MEDLINE, EMBASE, Cochrane Central Register of Controlled Trials, *meta*-Register (a clinical trials registry), and the references of included trials. The literature searches were conducted by an experienced librarian on 31 January 2012.

### Data items and collection process

The data abstracted included study characteristics, participant characteristics, and number of influenza cases per treatment group, confirmed by viral culture, PCR or serologic assay. A data abstraction form was developed and pilot tested. Two reviewers subsequently abstracted all of the data in duplicate. Discrepancies were resolved by discussion. Trial authors were contacted for data clarifications and additional unpublished data was received from six included published RCTs [10-15].

### Risk of bias appraisal

The Cochrane Risk of Bias seven-item tool was used to appraise the likelihood that the RCT results were affected by bias [16]. For the selective outcome reporting criterion, trial protocols were obtained and the outcomes reported in the protocol were compared to those reported in the final trial publication. For the other sources of bias criterion, industry-funded RCTs were scored as ‘unclear’, due to the potential for funding bias [17].

### Methodological issues

We also assessed other methodological issues identified *a priori* that are related to influenza and may have influenced the RCT results. These included case definitions used by health care workers to identify influenza-like illness (ILI), use of a surveillance system to monitor influenza cases, and randomization scheme across multisite trials. Case definition of influenza-like illness was defined according to the Centers for Disease Control and Prevention (CDC) as fever >37.8°C (100°F) with cough and/or sore throat [18]. Surveillance system was defined as a system to track ILI in the population and consisted of health care workers contacting participants to monitor ILI symptoms. Randomization scheme referred to the consistent use of an allocation schedule across trial study sites for multisite trials.

### Characterization of matched or mismatched vaccine strains

The process of characterizing matched versus mismatched strains was determined *a priori* and involved the two steps outlined below.

#### Step 1

Viral strains from influenza cases in the included RCTs were characterized through HI assays using vaccine strains as the reference. Influenza A strains from infected trial participants were matched with the strain in the vaccine if they belonged to the same A subtype (that is, H1N1 or H3N2) and were antigenically similar in the HI assay (that is, if they showed sufficient crossreaction in a HI chessboard table using ferret antisera; for example, with a HI typing quotient <fourfold titer). Influenza A viral strains were considered mismatched by antigenic drift if they were antigenically distinct from influenza A strains contained in the vaccine as per HI typing (for example, HI titer quotient ≥fourfold) or the characterization did not belong to a similar influenza A subtype contained in the vaccine (for example, H1N1 strains circulating but only H3N2 strains contained in the vaccine for bivalent vaccines with one H subtype).

For influenza B, the epidemiological situation is more complex. In recent years, there have been two coexisting phylogenetic influenza B lineages: B/Victoria and B/Yamagata [19,20]. Influenza B strains from infected trial participants were considered matched if the strain belonged to the same lineage and were antigenically similar to the vaccine strain as per HI typing (for example, HI typing quotient <fourfold titer). For influenza B mismatches, two different forms were considered. Mismatch by antigenic drift refers to strains of the same lineage that were antigenically distinct from influenza B strains contained in the vaccine as per HI typing (for example, HI quotient ≥fourfold titer), whereas mismatch by lineage refers to influenza B strains of different lineages. Whenever the influenza B lineage was not presented in the trial report, categorization was based on the influenza phylogenetic tree and verified by influenza experts on the team.

#### Step 2

When antigenic characterization of viral strains of infected participants was not reported, data from surveillance systems were used to determine circulating strains that occurred during the time and location of the trial conduct. These included WHO weekly epidemiological records, Mortality and Morbidity Reports Weekly (MMRW), Chinese National Influenza Center, and CDC influenza summary reports. These data were sometimes captured through correspondence with trial authors.

### Synthesis of included studies

The relative risk (RR) was calculated for the number of influenza cases per treatment group for included RCTs and 95% confidence intervals (CIs) were derived based on a normal approximation. Meta-analysis was conducted using a random effects model [21] for the live attenuated influenza vaccines (LAIV), trivalent inactivated vaccines (TIV), and other vaccines, separately. A *post hoc* sensitivity analysis was also conducted to examine the influence of categorizing the vaccines as being either an inactivated influenza vaccine or a live influenza vaccine. VE was derived based on pooled RRs using the following formula: (1 - RR) × 100%. Only influenza infections due to mismatched strains were included in the mismatched analysis, while only influenza infections due to matched strains were included in the matched analysis.

To assess for clinical and methodological heterogeneity, we examined the forest plots from the meta-analysis. The similarity between studies regarding participant and study characteristics was considered and subgroup analysis was conducted. Subgroup analyses determined *a priori* included age group (children <18 years of age, adults ≥18 years of age, older patients ≥65 years of age) and type of influenza (A versus B). Statistical heterogeneity was examined using the I^2^ statistic [22]. Funnel plots were performed to identify potential publication bias [23]. All analyses were conducted in SAS version 9.1 (SAS Inc., Cary, NC, USA).

## Results

### Literature search

**Figure 1:**
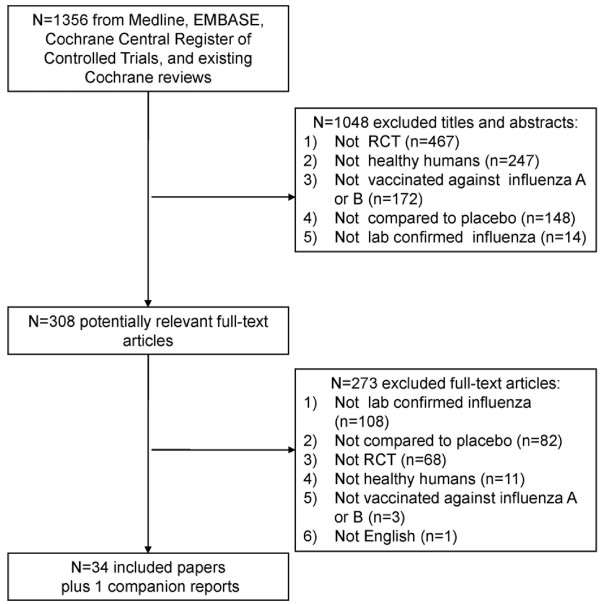
**Study flow.** This figure displays the flow of titles/abstracts and full-text studies through the systematic review.

The literature search resulted in a total of 1,356 citations, of which 308 were deemed potentially relevant (Figure 1). Reasons for exclusion at the full-text level of screening included not laboratory-confirmed influenza (108/273), no comparison to placebo (82/273), not a RCT (68/273), not a healthy population (11/273), not vaccinated against influenza A or B (3/273), and not disseminated in English (1/273). A total of 34 trials fulfilled the inclusion criteria [10-15,24-51] plus 1 companion report [52], which was used for supplementary data only (hence a total of 35 RCTs). In all, 32 RCTs [10-15,25-27,29-51] and 2 quasi-RCTs [24,28] provided data on 47 influenza seasons including 94,821 healthy participants.

### Trial characteristics

The trials were conducted between 1970 and 2009 (Table 1) in North America (22/34), Europe (3/34), Australia (1/34), Asia (3/34), and a mixture of multisite trials in North America, Europe, South America, Asia, and South Africa (4/34; Additional file 1). A total of 11 trials examined the TIV versus placebo [15,28,30,34,38,39,44-46,49],[50], 7 examined the LAIV versus placebo [10,35,37,40-43] and 6 examined both TIV and LAIV vaccines versus placebo [11,13,14,31,36,47]. Other vaccine types were examined in several RCTs, such as monovalent inactivated [24,25,51], bivalent attenuated [26,27], whole-virus vaccine [29], baculovirus-expressed HI influenza vaccine (rHAO) [12], and a trivalent recombinant HI protein vaccine [48]. One RCT examined both a TIV and bivalent vaccine compared to placebo [32].

**Table 1:** Antigenic characterization of viral strains

**Author (year)**	**Vaccine type**	**Vaccine composition**	**Type of laboratory-confirmed influenza used in the analysis**	**Circulating strains**	**Antigenic characterization as per author**	**Classification of laboratory-confirmed influenza viral strains as being matched**	**Classification of laboratory-confirmed influenza viral strains as being mismatched**
Leibovitz (1971) [24]	Other (MIV)	A/Hong Kong/Aichi/68 (H3N2)	Culture	A/Hong Kong/Aichi/68 (H3N2)	Typed by HI (cut-off: fourfold rise) to A/Hong Kong/Aichi/68, B/Massachusetts/66	A/Hong Kong/Aichi/68 (H3N2)	NA
Beutner (1979) [25]	Other (MIV)	A/Port Chalmers/1/73 (H3N2)	HI assay (secondary analysis)	A/Port Chalmers, A/Victoria strains	Typed by HI (cut-off: eightfold rise) to A/Port Chalmers and A/Victoria	A/Port Chalmers/1/73 (H3N2)	A/Victoria (H3N2, classified as antigenic drift)
Rytel (1977) [26]	Other (bivalent attenuated vaccine)	A/England/42/72 (H3N2), B/Hong Kong/5/72	Culture	A/Port Chalmers/1/73 (H3N2)	Typed by HI (cut-off: fourfold rise) to A/Hong Kong/8/66, A/England/42/72, A/Port Chalmers/1/73	NA	A/Port Chalmers/1/73 (H3N2) (reported as antigenic drift)
Monto (1982) [27]	Other (bivalent attenuated vaccine)	B/Ann Arbor/1/66, B/Hong Kong/8/73	HI assay (secondary analysis)	B/Singapore/79, B/Buenos Aires/79-like	Typed by HI (cut-off: fourfold rise) to B/Hong Kong (supplied by CDC) B/Tecumseh	NA	B/Singapore/79-like, B/Buenos Aires/79-like (reported as antigenic drift)
Tannock (1984) [28]	TIV	A/Brazil/11/78 (H1N1), A/Bangkok/I/79 (H3N2), B/Singapore/222/79	HI assay (secondary analysis)	A/H3N2 and influenza B strains were circulating (unspecified)	Typed by HI (cut-off: fourfold rise) to A/Bangkok/1/79	A/Bangkok/1/79 (H3N2)	NA
Keitel (1997) Y1^a^[29]; Keitel (1988) [52]	Other (WV)	A/Brazil/11/78 (H1N1), A/Philippines/2/82 (H3N2), B/Singapore/222/79	Culture	A/Victoria/7/83 (H1N1), B/USSR/100/83	Antigenic characterization not actively performed; based on predominant epidemic viruses	NA	A/Victoria/7/83 (H1N1) B/USSR/100/83 (classified as antigenic drift)
Keitel (1997) Y2^a^[29]; Keitel (1988) [52]	Other (WV)	A/Chile/1/83 (H1N1), A/Philippine/2/82 (H3N2), B/USSR/100/83	Culture	A/Philippines/2/82 (H3N2)	Antigenic characterization not actively performed; based on predominant epidemic viruses	A/Philippines/2/82 (H3N2)	NA
Keitel (1997) Y3^a^[29]; Keitel (1988) [52]	Other (WV)	A/Chile/1/83 (H1N1), A/Philippine/2/82 (H3N2), B/USSR/100/83	Culture	A/Mississippi/1/85 (H3N2), B/Ann Arbor/1/86	Antigenic characterization not actively performed; based on predominant epidemic viruses	NA	B/Ann Arbor/1/86 (classified as antigenic drift)
Keitel (1997) Y4^a^[29]; Keitel (1988) [52]	Other (WV)	A/Chile/1/83 (H1N1), A/Mississippi/1/85 (H3N2), B/Ann Arbor/1/86 (Victoria) plus A/Taiwan/1/86 (H1N1, monovalent supplement)	Culture	A/Taiwan/1/86 (H1N1)	Antigenic characterization not actively performed; based on predominant epidemic viruses	A/Taiwan/1/86 (H1N1)	NA
Keitel (1997) Y5^a^[29]; Keitel (1988) [52]	Other (WV)	A/Leningrad/360/86 (H3N2), A/Taiwan/1/86 (H1N1), B/Ann Arbor/1/86 (Victoria)	Culture	A/Sichuan/1/87 (H3N2)B/Victoria/2/87 (Victoria)	Antigenic characterization not actively performed; based on predominant epidemic viruses	NA	A/Sichuan/1/87 (H3N2), B/Victoria/2/87 (classified as antigenic drift
Gruber (1990) [30]	TIV	A/Chile/1/83 (H1N1), A/Philippines/2/82 (H3N2), B/USSR/100/83 (lineage unknown)	Culture or HI assay (secondary analysis)	B/Ann Arbor/1/86 (Victoria)	Typed by HI (cut-off: fourfold rise) to A/Chile, A/Philippines, A/Dunedin, A/Panama B/Ann Arbor, B/USSR	NA	B/Ann Arbor/1/86 (reported as antigenic drift)
Edwards (1994) Y1 [31]	LAIV, TIV	LAIV: A/Texas/1/85 (H1N1), A/Bethesda/1/85 (H3N2); TIV: A/Chile/1/83 (H1N1), A/Mississippi/1/85 (H3N2), B/Ann Arbor/1/86	Culture	A/Taiwan/1/86 (H1N1)	Typed by HI (cut-off: fourfold rise) by to the circulating strains and strains in the vaccine (supplied by CDC)	NA	A/Taiwan/1/86 (H1N1) (reported as antigenic drift)
Edwards (1994) Y2 [31]	LAIV, TIV	LAIV: A/Kawasaki/9/86 (H1N1), A/Bethesda/1/85 (H3N2); TIV: A/Taiwan/1/86 (H1N1), A/Leningrad/360/86 (H3N2), B/Ann Arbor/1/86	Culture	A/Sichuan/2/87 (H3N2), B/Victoria/2/87 (Victoria)	Typed by HI (cut-off: fourfold rise) by to the circulating strains and strains in the vaccine (supplied by CDC)	NA	A/Sichuan/2/87 (H3N2) (reported as antigenic drift)
Edwards (1994) Y3 [31]	LAIV, TIV	LAIV: A/Kawasaki/9/86 (H1N1), A/Los Angeles/2/87 (H3N2); other: A/Taiwan/1/86 (H1N1), A/Sichuan/2/87 (H3N2), B/Beijing/1/87	Culture	A/Taiwan/1/86 (H1N1), B/Yamagata/16/88 (Yamagata)	Typed by HI (cut-off: fourfold rise) by to the circulating strains and strains in the vaccine (supplied by CDC)	A/Taiwan/1/86 (H1N1)	NA
Edwards (1994) Y4 [31]	LAIV, TIV	LAIV: A/Kawasaki/9/86 (H1N1), A/Los Angeles/2/87 (H3N2); TIV: A/Taiwan/1/86 (H1N1), A/Shanghai/11/87 (H3N2), B/Yamagata/16/88	Culture	A/Shanghai/11/87 (H3N2)	Typed by HI (cut-off: fourfold rise) by to the circulating strains and strains in the vaccine (supplied by CDC)	A/Shanghai/11/87 (H3N2)	NA
Clover (1991) [32]	TIV, Other (Bivalent CR)	TIV: A/Chile/83 (H1N1), A/Mississippi/85 (H3N2), B/Ann Arbor/8 (Victoria); bivalent CR: A/Texas/1/85 (H1N1), A/Bethesda/1/85 (H3N2)	Viral culture or HI assay (secondary analysis)	A/Taiwan/1/86 (H1N1) closely related to A/Singapore/6/86 (H1N1), A/Chile/1/83 (H1N1)	Typed by HI (cut-off: eightfold rise or two ≥fourfold rises) to A/Chile and A/Taiwan	NA	A/Taiwan/1/86-like (H1N1)
Govaert (1994) [33]	Other (purified split viron vaccine)	A/Singapore/6/86 (H1N1), A/Beijing/353/89 (H3N2), B/Beijing/1/87 (Victoria), B/Panama/45/90 (Yamagata)	HI assay (secondary analysis)	A/Beijing/353/89 (H3N2), A/Singapore/6/86 (H1N1), B/Beijing/1/87 (Victoria), B/Panama/45/90 (Yamagata)	Antigenic characterization not actively performed; based on predominant epidemic viruses	A/Beijing/353/89 (H3N2)	NA
Powers (1995) [34]	TIV	A/Texas/36/91 (H1N1), A/Beijing/32/92 (H3N2), B/Panama/45/90 (Yamagata)	Culture or HI assay (secondary analysis)	A/Beijing/32/92 (H3N2)	Type by HI (cut-off: fourfold rise) to A/Beijing (supplied by CDC)	A/Beijing/32/92 (H3N2)	NA
Belshe (1998) [35]	LAIV	A/Texas/36/91-like (H1N1), A/Wuhan/359/95-like (H3N2), B/Harbin/7/94-like (Yamagata)	Culture	Influenza A (H3N2), influenza B. The isolation of influenza A or B among the study population paralleled that in the community in general.	Typed by HI (cut-off: fourfold rise) to A/Texas, A/Wuhan, B/Harbin	A/Wuhan/359/95-like (H3N2), B/Harbin/7/94-like (Yamagata)	NA
Rudenko (2001) [36]	LAIV, TIV	LAIV: A/Leningrad/134/17/57 (H3N2) or A/Texas/36/91(H1N1), A/Nanchang/933/95 (H3N2), B/Harbin/07/94 (Yamagata), B/Ann Arbor/1/86 (Victoria); TIV: A/Texas/36/91 (H1N1), A/Nanchang/933/95 (H3N2), B/Harbin/07/94 (Yamagata)	Culture or HI assay (secondary analysis)	Viral isolates similar to A/Texas/36/91 (H1N1), or B/Harbin/07/94	Typed by HI (cut-off: fourfold rise) to vaccine strains	A/Texas/36/91 (H1N1), B/Harbin/07/94 (Yamagata)	NA
Belshe (2000) [37]	LAIV	A/Shenzhen/227/95-like (H1N1), A/Wuhan/359/95 (H3N2), B/Harbin/7/94-like	Culture	A/Sydney/5/97-like; A/Wuhan/359/95-like and Influenza B	Typed by HI (cut-off: fourfold rise) to A/Nanchang (similar to A/Wuhan) and A/Sydney	A/Wuhan/359/95-like (H3N2)	A/Sydney/5/97-like (H3N2) (reported as antigenic drift)
Bridges (2000) Y1 [38]	TIV	A/Johannesburg/82/96 (H1N1), A/Nanchang/933/95 (H3N2), B/Harbin/7/94 (Yamagata)	Culture or HI assay (secondary analysis)	A/Sydney/5/97-like (H3N2), A/Michigan/8/98 (H3N2)	Typed by HI (cut-off: fourfold rise) to A/Johannesburg, A/Nanchang, B/Harbin, A/Sydney, A/Michigan (supplied by CDC)		A/Sydney/5/97-like (H3N2) (reported as antigenic drift)
Bridges (2000) Y2 [38]	TIV	A/Beijing/262/95 (H1N1), A/Sydney/5/97 (H3N2), and B/Harbin/7/94 (Yamagata)	Culture or HI assay (secondary analysis)	A/Sydney/5/97-like (H3N2), B/Beijing/184/93-like (Yamagata)	Typed by HI (cut-off: fourfold rise) to A/Johannesburg, A/Nanchang, B/Harbin, A/Sydney, A/Michigan (supplied by CDC)	A/Sydney/5/97-like (H3N2) B/Beijing/184/93-like (Yamagata)	NA
Hoberman (2003) Y1 [39]	TIV	A/Beijing/262/95 (H1N1), A/Sydney/15/97 (H3N2), and B/Yamanashi/166/98 (Yamagata)	Culture	A/Beijing/262/95 (H1N1), A/Sydney/15/97 (H3N2)	Typed by HI (cut-off: fourfold rise) to A/Beijing and A/Sydney	A/Beijing/262/95 (H1N1), A/Sydney/15/97 (H3N2)	NA
Hoberman (2003) Y2 [39]	TIV	A/New Caledonia/20/99 (H1N1), A/Panama/07/99 (H3N2), and B/Yamanashi/166/98 (Yamagata)	Culture	A/New Caledonia/20/99 (H1N1), A/Panama/07/99 (H3N2) B/Yamanashi/166/98 (Yamagata)	Typed by HI (cut-off: fourfold rise) to A/New Caledonia, B/Yamanashi, A/Panama	A/New Caledonia/20/99 (H1N1), A/Panama/07/99 (H3N2), B/Yamanashi/166/98 (Yamagata)	NA
Tam (2007) Y1 [40]	LAIV	A/New Caledonia/20/99 (H1N1), A/Sydney/05/97 (H3N2), B/Yamanashi/166/98 (Yamagata)	Culture	B/Sichuan/379/99 (Yamagata), B/Hong Kong/330/01-like (Victoria) (reported in Belshe review [59])	Typed by HI (cut-off: fourfold rise) to A/New Caledonia, A/Sydney, B/Yamanashi (supplied by the CDC)	Influenza A strains antigenically similar to vaccine (strains unspecified)	B/Sichuan-like (reported as antigenic drift), B/Hong Kong-like (classified as lineage mismatch as per Belshe review [59])
Tam (2007) Y2 [40]	LAIV	A/New Caledonia/20/99 (H1N1), A/Panama/2007/99 (H3N2), and B/Yamanashi/166/98 (Yamagata)	Culture	B/Victoria/504/00 (Victoria lineage) (B/Sichuan/379/99-like) virus (Yamagata)	Typed by HI (cut-off: fourfold rise) to A/New Caledonia/20/9 A/Sydney/5/97, B/Victoria/2/87 (supplied by CDC)	NA	B/Sichuan-like (reported as antigenic drift), B/Hong Kong-like (classified as lineage mismatch as per Belshe review [59])
Vesikari (2006) Y1 [41]	LAIV	A/New Caledonia/20/99 (H1N1), A/Sydney/05/97 (H3N2), B/Yamanashi/166/98 (Yamagata)	Culture	A/New Caledonia/20/99-like (H1N1) A/Panama/07/99-like (H3N2) B/Sichuan/379/99-like (Yamagata)	Typed by HI (cut-off NR) to vaccine strains and community acquired strains	A/New Caledonia/20/99-like (H1N1), A/Panama/07/99-like (H3N2)	Influenza A strains (unspecified; reported as antigenic drift), B/Sichuan-like (classified as antigenic drift as per Belshe review [59]), influenza B strains (unspecified; reported as lineage mismatch)
Vesikari (2006) Y2 [41]	LAIV	A/New Caledonia/20/99 (H1N1), A/Panama/07/99 (H3N2), B/Victoria/504/00 (Yamagata)	Culture	A/New Caledonia/20/99-like (H1N1), A/Panama/07/99-like (H3N2), B/Hong Kong/330/01-like (Victoria)	Typed by HI (cut-off NR) to vaccine strains and community acquired strains	A/New Caledonia/20/99-like (H1N1), A/Panama/2007/99-like (H3N2), B/Victoria/504/00-like (Yamagata)	B/Hong Kong-like (reported as lineage mismatch)
Forrest (2008) [42]	LAIV	A/New Caledonia/20/99-like (H1N1), A/Panama strain (H3N2), B/Victoria/504/00 (Yamagata)	Culture	A/New Caledonia/20/99 (H1N1), A/Fujian/411-02-like, B/Hong Kong/330/01-like (Victoria)	Typed by HI (cut-off: fourfold rise) to vaccine strains and community acquired strains	Antigenically similar strains to H3N2 (unspecified)	Influenza B strains (unspecified; reported as lineage mismatch)
Bracco Neto (2009) Y1^b^[10]	LAIV	A/New Caledonia/20/99 -like (H1N1), A/Moscow/10/99-like (H3N2), B/Sichuan/379/99-like (Yamagata)	Culture	A/New Caledonia/20/99 (H1N1), A/Panama/07/99-like (H3N2), B/Sichuan/379/99-like (Yamagata)	Typed by HI (cut-off: fourfold rise) to vaccine strains and community acquired strains (unspecified)	A/New Caledonia/20/99-like (H1N1), A/Panama/2007/99-like (H3N2), B/Yamanashi/166/98-like (Yamagata), B/Victoria/504/00-like (Yamagata)	Influenza A strains (unspecified; reported as antigenic drift), influenza B strains (unspecified; reported as lineage mismatch)
Bracco Neto (2009) Y2^b^[10]	LAIV	A/New Caledonia/20/99 (H1N1) A/Moscow/10/99 (H3N2), B/Hong Kong/330/01-like (Victoria)	Culture	A/New Caledonia/20/99 (H1N1), A/Panama/07/99-like (H3N2) B/Hong Kong/330/01-like (Victoria)	Typed by HI (cut-off: fourfold rise) to vaccine strains and community acquired strains (unspecified)	A/New Caledonia/20/99-like, A/Panama/07/99-like, B/Victoria-like (Victoria)	B/Hong Kong (reported as lineage mismatch)
Lum (2010) [43]	LAIV	A/New Caledonia/20/99 (H1N1), A/Panama/07/99 (H3N2), B/Hong Kong/330/2001 (Victoria)	Culture	A/New Caledonia/20/99-like (H1N1), A/Panama/07/99-like (H3N2), A/Fujian/411-02-like (H3N2) in Asian regions, B/Victoria-like (Victoria)	Typed by HI (cut-off: fourfold rise) and PCR sequencing to A/Fujian/411/02-like, B/Victoria/504/00-like, A/New Caledonia/20/99-like, A/Panama/2007/99-like, B/Hong Kong/1351/02-like (supplied by CDC and Wyeth)	A/New Caledonia-like (H1N1), A/Panama-like (H3N2), B/Hong Kong-like (Victoria)	A/Fujian-like and B/Victoria-like (Victoria) (report as antigenic drift)
Langley (2011) [44]	TIV	A/New/Caledonia/20/99 (H1N1), A/Panama/07/99 (H3N2) B/Shangdong/7/97 (Victoria)	Culture	A/New Caledonia/20/99 (H1N1)-like. A/Fujian (H3N2), B/Hong Kong/330/01-like (Victoria), B/Sichuan/379/99-like (Yamagata)	Typed by HI (cut-off: fourfold rise) (not specified)		A/Fujian (reported as antigenic drift accounting for 96.8% of H3N2 isolates)
Ohmit (2006)^b^[11]	LAIV, TIV	LAIV: A/New Caledonia/20/99 (H1N1), A/Wyoming/03/03-like (H3N2), B/Jilian/20/03-like (Yamagata lineage); TIV: A/New Caledonia/20/99 (H1N1), A/Wyoming/3/03 (H3N2), B/Jiangsu/10/03-like (Yamagata lineage)	Culture	A/California/07/04-like, A/Wisconsin-like (H3N2), B/Hawaii/33/04-like (Victoria lineage)	Typed by HI (cut-off: fourfold rise) to vaccine strains and A/California/07/04, B/Hawaii/33/04-like (supplied by the CDC)	B/Shanghai/361/02-like (Yamagata)	A/California/07/04-like (H3N2) (report as antigenic drift), B/Hawaii/33/04-like (report as lineage mismatch)
Treanor (2007)^b^[12]	Other (purified rHAO vaccine)	A/New Caledonia/20/99 (H1N1), A/Wyoming/3/03 (H3N2), B/Jiangsu/10/03 (Yamagata lineage)	Culture	A/California/7/04 -like (H3N2), A/Wisconsin-like (H3N2), B/Yamagata/16/88-like (Yamagata)	Typed by HI (cut-off: fourfold rise) to vaccine and circulating strains (unspecified) (supplied by CDC)	NA	A/California-like (H3N2, report as antigenic drift), B/Yamagata-like (report as lineage mismatch)
Beran (2009) [45]	TIV	A/New Caledonia/20/99 IVR-116 virus (H1N1), A/New York/55/04 X-157 (H3N2) B/Jiangsu/10/03 (Yamagata lineage)	Culture	A/New Caledonia/20/99 (H1N1), A/California,//07/04-like, B/Jiangsu/10/2003-like viruses (Yamagata lineage)	Typed by HI (cut-off: fourfold rise) to unspecified strains	A/California-like (H3N2), A/New Caledonia-like (H1N1), A/Czech Republic-like (H1N1)	B/Hong Kong-like, (reported as lineage mismatch)
Jackson (2010) Y1 [15]	TIV	A/New Caledonia/20/99 (H1N1), A/New York/55/04 (H3N2), B/Jiangsu/10/03 (Yamagata lineage)	Culture	A/New Caledonia/20/99 A (H1N1), A/California/07/04-like (H3N2), A/Wisconsin-like (H3N2), B/Shanghai/361/02 (Yamagata), B/Florida/07/04-like (lineage unknown) B/Ohio/1/05 (Victoria)	Typed by HI (cut-off: fourfold rise) to vaccine and circulating strains (unspecified) (supplied by CDC and WHO)	Report results for influenza A and B strains (unspecified) with ≤fourfold difference in HI titer compared to vaccine strains	Report results for influenza A and B strains (unspecified) with >fourfold difference in HI titer compared to vaccine strains
Jackson (2010) Y2 [15]	TIV	A/New Caledonia/20/99 (H1N1), A/Wisconsin/67/05 (H3N2), B/Malaysia/2506/04 (Victoria lineage)	Culture	A/New Caledonia/20/99, A (H1N1), A/Solomon Islands/3/06 A (H1N1), A/Wisconsin/67/05 A (H3N2), B/Yamagata/16/88 (Yamagata) B/Ohio/01/05 (Victoria)	Typed by HI (cut-off: fourfold rise) to vaccine and circulating strains (unspecified) (supplied by CDC and WHO)	Report results for influenza A and B strains (unspecified) with ≤fourfold difference in HI titer compared to vaccine strains	Report results for influenza A and B strains (unspecified) with >fourfold difference in HI titer compared to vaccine strains
Ohmit (2008)^b^[13]	LAIV, TIV	LAIV/TIV: A/New Caledonia/20/99 (H1N1), A/New York/55/04 (H3N2), B/Jiangsu/10/03 (Yamagata)	Culture or PCR	A/California/07/04-like (H3N2), A/Wisconsin/67/05 (H3N2), B/Shanghai/361/02 (Yamagata), B/Florida/07/04-like (Yamagata), B/Ohio/01/05 (Victoria)	Typed by HI (cut-off: fourfold rise) to A/Wisconsin/67/05, B/Ohio/01/05, and vaccine strains	A/California/07/04 (H3N2)	B/Ohio/01/05-like (reported as lineage mismatch)
Beran (2009) [46]	TIV	A/New Caledonia/20/99 IVR-116 (H1N1), A/Wisconsin/67/05 (H3N2), B/Malaysia/2506/04 (Victoria)	Culture	A/New Caledonia/20/99, A/Wisconsin/67/05, B/Yamagata-like (Yamagata)	Typed by HI (cut-off: fourfold rise) to unspecified strains (supplied by CDC and WHO)	A/Wisconsin (H3N2)	A/H1N1 strains (unspecified; reported as antigenic drift), B/Yamagata-like (reported as lineage mismatch)
Monto (2009)^b^[14]	LAIV, TIV	LAIV/TIV: A/Solomon Islands/3/06 (H1N1), A/Wisconsin/67/05 (H3N2), B/Malaysia/2506/04 (Victoria)	PCR	A/Brisbane/59/07-like (H1N1), A/Wisconsin/67/05 (H3N2)	Typed by HI (cut-off: eightfold rise) to vaccine strains and circulating strains (supplied by CDC)	A/Wisconsin-like (H3N2)	A/Brisbane-like (report as antigenic drift), B/Yamagata-like (reported as lineage mismatch)
Frey (2010) [47]	LAIV, TIV	LAIV/TIV: A/Solomon Islands/3/06 (H1N1)-like, A/Wisconsin/67/05 (H3N2)-like, B/Malaysia/2506/04-like (Victoria)	Culture	A/Solomon Islands/3/06 (H1N1) A/Brisbane/59/07-like (H3N2) A/Wisconsin/67/05-like (H3N2), B/Florida/4/06 (Yamagata), B/Ohio/01/05 (Victoria), B/Malaysia/2506/04 (Victoria)	Typed by HI (cut-off: fourfold rise) to unspecified strains (supplied by CDC)	Report results for influenza A (H1N1, H3N2) and B strains (unspecified) with ≤fourfold difference in HI titer compared to vaccine strain	Report results for influenza A (H1N1, H3N2) and B strains (unspecified) with >fourfold difference in HI titer compared to vaccine strain
Treanor (2011) [48]	Other (FluBlok®)	A/Solomon Island/3/06 (H1N1), A/Wisconsin/67/05 (H3N2), B/Malaysia/2506/04 (Victoria)	Culture	A/Brisbane/59/07 (H1N1), A/Brisbane/10/07 (H3N2), B/Florida/04/06 (Yamagata)	Typed by HI (cut-off: fourfold rise) to strains (unspecified)	A/Wisconsin/67/05-like (H3N2)	A/Brisbane/59/07-like (H1N1) (reported as antigenic drift), B/Florida/04/06 (reported as lineage mismatch) (data NR by treatment group)
Barrett (2011) [49]	TIV	A/Brisbane/59/07 (H1N1), A/Uruguay/716/07, B/Florida/4/06 (Yamagata)	Culture or RT-PCR	A/Brisbane/59/07 B/Florida/04/06 (Yamagata) B/Victoria/02/87 (Victoria)	Typed by HI (lowest titer at which the sum of the specificity and sensitivity was maximum) to strains (unspecified) (supplied by CDC)	A/Brisbane/59/07 (H1N1), A/Uruguay/716/07 (H3N2), B/Florida/4/06	Influenza A strains (unspecified), B/Victoria-like (reported as lineage mismatch)
Cowling (2010) [50]	TIV	A/Brisbane/59/07 (H1N1)-like, A/Brisbane/10/07 (H3N2)-like, and B/Florida/4/06 (Yamagata)	RT-PCR	A/Perth/16/09-like (H3N2)	Typed by HI (cut-off: fourfold rise) to A/Brisbane (H1N1), A/Brisbane (H3N2), A/California, A/Perth, H1N1 and B/Florida	A/Brisbane (H1N1), A/Brisbane (H3N2), A/California, and B/Florida	A/Perth/16/09-like (H3N2) (report as antigenic drift) and pandemic A/H1N1
Talaat (2010) [51]	Other (MIV)	A/H1N1/2009	RT-PCR	H1N1	Typed by HI (cut-off: fourfold rise) to A/H1N1/09	NA	A/H1N1 (HIN1)

Circulating strains refers to the influenza viral strains circulating in the specified time and region as reported in the publication. Circulating viral strains were crossreferenced with WHO weekly epidemiological records, Chinese National Influenza Centre CDC influenza summary reports, Mortality and Morbidity Reports Weekly (MMRW, published by CDC), where applicable. In all cases circulating strains matched influenza documentation as noted above. Antigenic characterization as per author refers to how the author determined and classified influenza strains in participants with laboratory-confirmed influenza. Immunogenicity to vaccine and non-vaccine strains conducted using cut-off values to determine match or mismatch between strains contained in the vaccine and those that were circulating. Classification of laboratory-confirmed influenza viral strains as being matched refers to the categorization of influenza strains that are antigenically similar or well matched to vaccine strains. Strains listed in the column represent break through strains that despite being well matched to vaccine caused influenza infections in the study population. In some cases, antigenically similar or well matched strains were presented in the study and are listed in the table as ‘reported’. In other cases, antigenically similar or well matched strains was determined by surveillance data as documented above, in these cases the strains are listed as ‘classified as’. Classification of laboratory-confirmed influenza viral strains as being mismatched refers to the categorization of influenza strains that are antigenically distinct or mismatched to vaccine strains. Strains listed in the column represent break through strains that were not protected through vaccination. In some cases, antigenically distinct or mismatched strains were presented in the study by authors and are listed in the table as ‘reported’. In other cases, antigenically distinct or mismatched strains was determined by subtracting well matched strains from any strains reported in the publications, these strains are listed as ‘classified as’ by authors of this review. Influenza B mismatched strains are further classified as antigenic drifts or lineage drifts. Antigenic drift represents viral strains that have mutated but are still classified within the same lineage. Lineage drifts refer to the distinction between Yamagata and Victoria lineages of influenza B. Classification of lineage was either reported by author (denoted in table as ‘reported’) or categorized based on phylogenic tree of lineages (denoted as ‘classified’).

^a^Main publication.

^b^Unpublished data was obtained from the author.

*CDC* Center for Disease Control and Prevention, *HI* haemagglutinin inhibition, *LAIV* live attenuated influenza vaccine, *MIV* monovalent inactivated vaccine, *NA* not applicable, *NR* not reported, *RT-PCR* reverse transcriptase polymerase chain reaction, *TIV* trivalent influenza vaccine, *WHO* World Health Organization, *WV* whole viral vaccine.

In all, 22 RCTs (including information on 34 influenza seasons) provided data on matched influenza strains [10,11,13-15,24,29,31,35,37],[39-43,45-51], while 20 RCTs (including data from 33 influenza seasons) provided data on mismatched influenza strains [10-15,26,29,31,37,40-47,49,50] (Table 1). Influenza infection was confirmed by culture in 20 RCTs [10-12,15,24,26,29,31,35,37],[39-48], PCR in 3 RCTs [14,50,51], and culture or PCR in 2 RCTs [13,49]. Nine RCTs included serologic assays to determine influenza infection; these were included in the secondary analysis only [25,27,28,30,32-34,36,38].

### Participant characteristics

The RCTs included data on a total of 94,821 participants (Table 2). A total of 13 of the RCTs were conducted among children [10,25,30,32,35,37,39-43,49],[50], and 2 were conducted among seniors [33,36]. One RCT included children (15%) and adults (85%) [31], and another included adults and seniors [51]; the remaining RCTs were conducted among adults only. The percentage of female participants ranged from 22% [38] to 100% [26].

**Table 2:** Patient characteristics

**Lead author (year)**	**Country of conduct and year**	**Age category**	**Mean age (SD) in years**	**M/F, %**	**Sample size**	
					**Vaccine**	**Placebo**
Leibovitz (1971) [24]	USA, 1970	Adults	NR	NR	1,682	7,934
Beutner (1979) [25]	USA, 1974	Children	Range: 7 to 14	50/50	520	460
Rytel (1977) [26]	USA, 1974	Adults	NR	0/100	95	48
Monto (1982) [27]	USA, 1979	Adults	NR	NR	144	140
Tannock (1984) [28]	Australia, 1981	Adults	34.8 (13.9)	69/31	19	20
Keitel (1997) [29]	USA, 1983 to 1988	Adults	Range: 30 to 60	NR	Y1: 161	Y1: 298
					Y2: 172	Y2: 241
					Y3: 153	Y3: 253
					Y4: 203	Y4: 217
					Y5: 121	Y5: 145
Gruber (1990) [30]	USA, 1985	Children	7.9 (3.3)	NR	54	77
Edwards (1994) [31]	USA, 1986 to 1990	Adults/Children	Range: 1 to 65	NR	LAIV/TIV	
					Y1: 872/878	Y1: 878
					Y2: 1,029/1,060	Y2: 1,064
					Y3: 1,114/1,126	Y3: 1,125
					Y4: 999/1,016	Y4: 1,016
Clover (1991) [32]	USA, 1989	Children	8.8 (3.6)	NR	TIV/BIV 54/56	82
Govaert (1994) [33]	The Netherlands, 1991	Older patients	Range: 60 to 91	47/53	927	911
Powers (1995) [34]	USA, 1993	Adults	Range: 18 to 45	NR	TIV/other 26/26	24
Belshe (1998) [35]	USA, 1996	Children	3.5 (1.4)	47/53	1,070	532
Rudenko (2001) [36]	Russia, 1996	Older patients	Median: 73, Range: 41 to 95	30/70	LAIV/TIV 111/93	109
Belshe (2000) [37]	USA, 1997	Children	4.5 (1.4)	52/48	917	441
Bridges (2000) [38]	USA, 1997 to 1999	Adults	Median: 43.5	78/22	Y1: 138	Y1: 137
					Y2: 141	Y2: 137
Hoberman (2003) [39]	USA, 1999 to 2001	Children	Range: 0.5 to 2	56/44	Y1: 273	Y1: 138
					Y2: 252	Y2: 123
Tam (2007) [40]	Multisite trial in Asia, 2000 to 2002	Children	1.9 (0.6)	53/47	Y1: 1,653	Y1: 1,111
					Y2: 503	Y2: 494
Vesikari (2006) [41]	Multisite trial in Europe and Israel, 2000 to 2001	Children	2.0 (0.7)	51/49	Y1: 951	Y1: 665
					Y2: 640	Y2: 450
Bracco Neto (2009)^a^[10]	Multisite trial in South Africa and South America, 2001 to 2002	Children	Range: 0.5 to 3	49/51	Y1: 944	Y1: 942
					Y2: 338	Y2: 342
Forrest (2008) [42]	Multisite in Asia, 2002	Children	1.8 Range: 0.5 to 3	NR	525	516
Lum (2010) [43]	Multisite trial in Asia, Europe and South America, 2002	Children	1.2 (0.3)	50/50	765	385
Langley (2011) [44]	Canada, 2003	Adults	37.1 (12.2)	46/54	455	443
Ohmit (2006)^a^[11]	USA, 2004	Adult	26.9 (9.3)	38/62	LAIV/TIV 519/522	206
Treanor (2007)^a^[12]	USA, 2004	Adults	Median: 31, Range: 18 to 49	37/63	151	153
Beran (2009) [45]	Czech Republic, 2005	Adults	35 (13)	45/55	4,137	2,066
Jackson (2010) [15]	USA, 2005	Adults	32.7 (9.1)	40/60	Y1: 1,706	Y1: 1,725
					Y2: 2,011	Y2: 2,043
Ohmit (2008)^a^[13]	USA, 2005	Adults	24.9 (NR)	40/60	LAIV/TIV 853/867	338
Beran (2009) [46]	Multisite trial Europe, 2006	Adults	40.0 (13.3)	40/60	5,103	2,549
Monto (2009)^a^[14]	USA, 2007	Adults	23.3 (7.4)	38/62	LAIV/TIV 813/814	325
Frey (2010) [47]	Multisite trial North America and Europe, 2007	Adults	32.5 (NR)	44/45	LAIV/TIV 3,776/3,638	3,843
			Range: 18 to 48			
Treanor (2011) [48]	USA, 2007	Adults	32.5(NR)	41/59	2,344	2,304
			Range: 18 to 55			
Barrett (2011) [49]	Multisite trial in USA, 2008	Children	Range: 18 to 49	NR	3,619	3,617
Cowling (2010) [50]	Hong Kong, 2008	Children	Range: 6 to 15	53/47	71	48
Talaat (2010) [51]	USA, 2009	Adults and older patients	56.5 (18.0)	43/57	389	97

^a^Unpublished data was obtained from the author(s).

*LAIV* live attenuated influenza vaccine, *NR* not reported, *TIV* trivalent inactivated vaccine.

### Risk of bias

**Figure 2:**
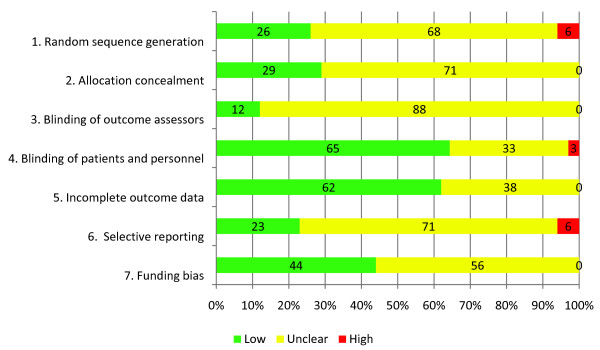
**Risk of bias across all studies.** This figure represents the risk of bias presented in the included studies. Green refers to a low risk of bias, yellow refers to an unclear risk of bias, and red refers to a high risk of bias.

Of the included trials, only 26% adequately reported sequence generation and 29% adequately reported allocation concealment (Figure 2). The majority of the RCTs blinded participants and physicians (65%) and addressed incomplete data (62%). Only half of the RCTs were deemed to be free from other sources of bias (44%; 56% scored unclear because they were industry funded) and 23% were free from selective outcome reporting (Table 3).

**Table 3:** Risk of bias

**Lead author (year)**	**Country of conduct and year**	**Adequate sequence generation**	**Allocation concealment**	**Blinding outcome assessors**	**Blinding patients and providers**	**Incomplete outcome data addressed**	**Free of selective reporting**	**Funding source**
Leibovitz (1971) [24]	USA, 1970	High	Unclear	Unclear	Unclear	Low	Unclear	Low
Beutner (1979) [25]	USA, 1974	Unclear	Unclear	Unclear	Unclear	Unclear	Unclear	Low
Rytel (1977) [26]	USA, 1974	Low	Low	Unclear	Unclear	Low	Unclear	Unclear
Monto (1982) [27]	USA, 1979	Unclear	Unclear	Unclear	Unclear	Low	Unclear	Low
Tannock (1984) [28]	Australia, 1981	High	Unclear	Unclear	Low	Low	Unclear	Unclear
Keitel (1997) [29]	USA, 1983 to 1988	Unclear	Unclear	Unclear	Unclear	Unclear	Unclear	Low
Gruber (1990) [30]	USA, 1985	Unclear	Unclear	Unclear	Low	Unclear	Unclear	Low
Edwards (1994) [31]	USA, 1986 to 1990	Low	Low	Unclear	Low	Unclear	Unclear	Low
Clover (1991) [32]	USA, 1989	Unclear	Unclear	Unclear	Unclear	Unclear	Unclear	Low
Govaert (1994) [33]	The Netherlands, 1991	Unclear	Low	Unclear	Low	Low	Unclear	Low
Powers (1995) [34]	USA, 1993	Unclear	Unclear	Unclear	Low	Unclear	Unclear	Low
Belshe (1998) [35]	USA, 1996	Unclear	Unclear	Unclear	Low	Unclear	Unclear	Low
Rudenko (2001) [36]	Russia, 1996	Unclear	Unclear	Unclear	Unclear	Unclear	Unclear	Unclear
Belshe (2000) [37]	USA, 1997	Unclear	Unclear	Unclear	Low	Unclear	Unclear	Low
Bridges (2000) [38]	USA, 1997 to 1998	Unclear	Unclear	Unclear	Low	Low	Unclear	Low
Hoberman (2003) [39]	USA, 1999 to 2000	Low	Unclear	Low	High	Low	Unclear	Unclear
Tam (2007) [40]	Multisite trial in Asia, 2000 to 2001	Low	Low	Unclear	Low	Unclear	Unclear	Unclear
Vesikari (2006) [41]	Multisite trial in Europe and Israel, 2000 to 2001	Unclear	Unclear	Unclear	Low	Low	Unclear	Unclear
Bracco Neto (2009)^a^[10]	Multisite trial in South Africa and South America, 2001 to 2002	Low	Low	Low	Unclear	Unclear	Unclear	Unclear
Lum (2010) [43]	Multisite trial in Asia, Europe and South America, 2002	Unclear	Low	Low	Low	Low	Unclear	Unclear
Forrest (2008) [42]	Multisite in Asia, 2002	Unclear	Unclear	Unclear	Low	Unclear	Unclear	Unclear
Langley (2011) [44]	Canada, 2003	Unclear	Unclear	Low	Low	Low	Unclear	Unclear
Ohmit (2006)^a^[11]	USA, 2004	Unclear	Unclear	Unclear	Low	Low	Low	Low
Treanor (2007)^a^[12]	USA, 2004	Unclear	Unclear	Unclear	Low	Low	Low	Unclear
Beran (2009) [45]	Czech Republic, 2005	Low	Low	Unclear	Low	Low	High	Unclear
Jackson (2010) [15]	USA, 2005	Low	Low	Unclear	Unclear	Low	Low	Unclear
Ohmit (2008)^a^[13]	USA, 2005	Unclear	Unclear	Unclear	Low	Low	Low	Low
Beran (2009) [46]	Multisite trial Europe, 2006	Unclear	Unclear	Unclear	Low	Low	High	Unclear
Monto (2009)^a^[14]	USA, 2007	Unclear	Unclear	Unclear	Low	Low	Low	Unclear
Frey (2010) [47]	Multisite trial North America and Europe, 2007	Unclear	Unclear	Unclear	Unclear	Low	Low	Unclear
Treanor (2011) [48]	USA, 2007	Unclear	Unclear	Unclear	Unclear	Low	Low	Unclear
Barrett (2011) [49]	Multisite trial in USA, 2008	Low	Low	Unclear	Low	Unclear	Unclear	Unclear
Cowling (2010) [50]	Hong Kong, 2008	Low	Low	Unclear	Low	Low	Low	Low
Talaat (2010) [51]	USA, 2009	Unclear	Unclear	Unclear	Low	Low	Unclear	Unclear

^a^Unpublished data was obtained from the author(s).

The trials were conducted over 40 years and the risk of bias for older studies was compared to newer studies, using 1991 as the midpoint. No trends were observed for sequence generation or allocation sequence and differences between groups were minimal. Blinding of outcome assessors and the reporting of incomplete data improved in recent studies by 17%. Furthermore, selective outcome reporting in recent studies showed an improvement of 42% in the risk of bias. There was also an increase in the proportion of RCTs scoring ‘unclear’ on other risk of bias criterion, as more trials were funded by private industry in recent years.

### Methodological issues

Only 15% of trials were free of other methodological issues, which included adequate information on case definition of ILI and surveillance of influenza, as well as details regarding randomization across multiple sites [12,38,44,48,50] (Additional file 2). Almost half of trials (16 of 34) did not adequately report a case definition of ILI and the remaining studies were unclear in at least 1 of the domains for other methodological concerns.

### LAIV versus placebo

The results from all LAIV versus placebo meta-analyses are presented in Additional file 3. For simplicity, we have focused on the results specific to children aged <18 years for the primary outcome.

**Figure 3:**
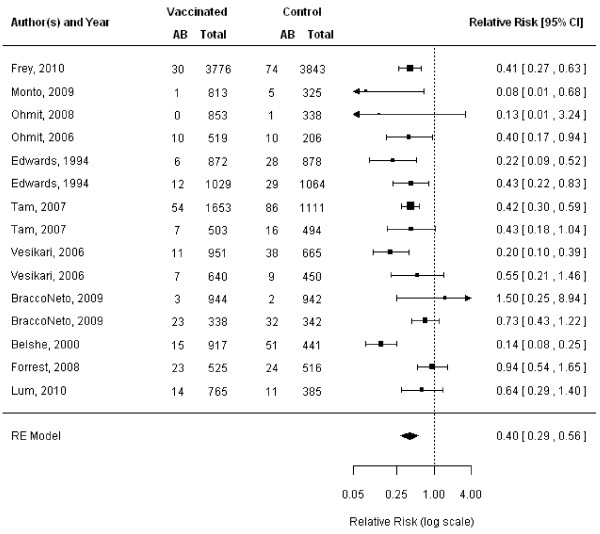
**Meta-analysis of live attenuated influenza vaccine (LAIV), mismatched.** This figure represents the relative risk (RR) of an influenza infection occurring when the circulating strain does not match strains contained in the LAIV.

**Figure 4:**
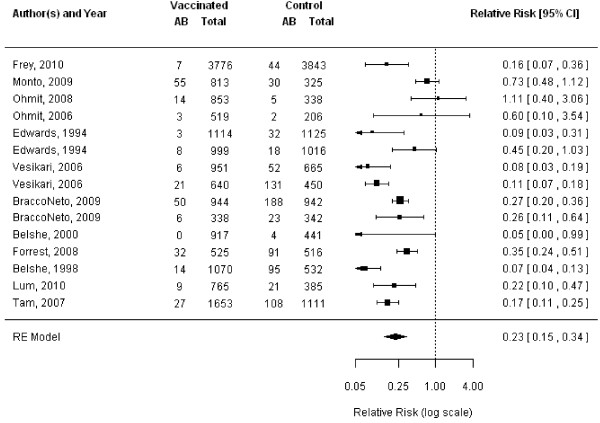
**Meta-analysis of live attenuated influenza vaccine (LAIV), matched.** This figure represents the relative risk (RR) of an influenza infection occurring when the circulating strain matches strains contained in the LAIV.

The LAIV was protective against mismatched strains overall (11 RCTs, VE 60%, 95% CI 44% to 71%; Figure 3; Additional file 3) [10,11,13,14,31,37,40-43,47], as well as among children aged 6 to 36 months (6 RCTs, VE 54%, 95% CI 28% to 71%) [10,37,40-43] when strains were mismatched. Similarly, the LAIV was effective in protecting against infection when circulating strains were well matched overall (12 RCTs, VE 77%, 95% CI 67% to 86%, Figure 4) [10,11,13,14,31,35,37,40-43],[47]. LAIV provided significant protection among children when circulating strains matched vaccine composition (seven RCTs, VE 83%, 95% CI 75% to 88%) [10,35,37,40-43].

When influenza A strains did not match LAIV composition, protection against infection was statistically significant among children (five RCTs, VE 75%, 95% CI 41% to 90%) [10,37,40,41,43]. LAIV was also effective in protecting against influenza A when strains were well matched in children (six RCTs, VE 84%, 95% CI 76% to 90%) [10,35,40-43].

When influenza B strains were mismatched, LAIV was effective among children (five RCTs, VE 42%, 95% CI 22% to 56%) [10,40-43]. Furthermore, LAIV was more protective against drifted B strains (two RCTs, VE 62%, 95% CI 21% to 81%) [40,41] than lineage mismatch for influenza B (five RCTs, VE 34%, 95% CI 4−% to 59%) [10,40-43] in children. Similarly, LAIV was effective against influenza B when circulating strains matched vaccine strains in children (four RCTs, VE 79%, 95% CI 58% to 90%) [10,35,41,43].

### TIV versus placebo

The results from all TIV versus placebo meta-analyses are presented in Additional file 3. For simplicity in the text, we have focused on the results specific to adults aged ≥18 years.

**Figure 5:**
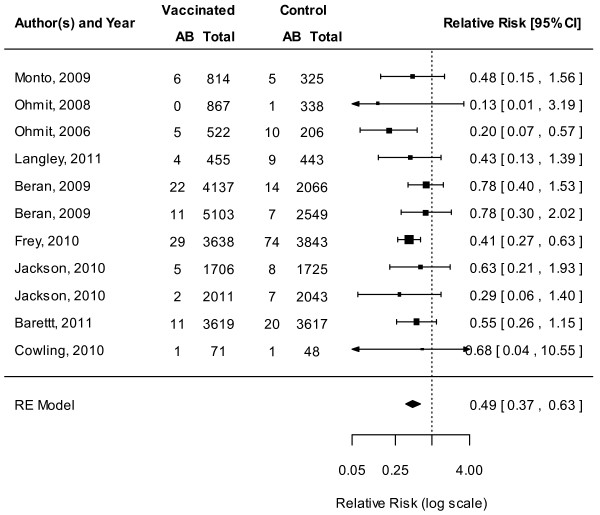
**Meta-analysis of trivalent inactivated vaccine (TIV), mismatched.** This figure represents the relative risk (RR) of an influenza infection occurring when the circulating strain does not match strains contained in the TIV.

**Figure 6:**
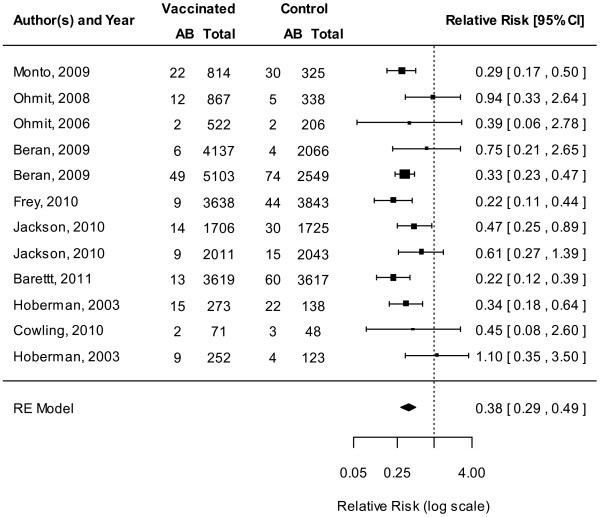
**Meta-analysis of trivalent inactivated vaccine (TIV), matched.** This figure represents the relative risk (RR) of an influenza infection occurring when the circulating strain does not match strains contained in the TIV.

The TIV showed protection against mismatched strains overall (11 RCTs, VE 56%, 95% CI 43% to 66%, Figure 5) [11,13-15,31,44-47,49,50] and among adults (9 RCTs, VE 52%, 95% CI 37% to 63%) [11,13-15,44-47,49]. Similarly, TIV vaccines showed protection against matched strains overall (11 RCTs, VE 65%, 95% CI 58% to 72%, Figure 6) [11,13-15,31,39,45-47,49,50] and among adults (8 RCTs, VE 65%, 95% CI 54% to 73%) [11,13-15,45-47,49].

In adults, TIV provided significant protection against mismatched influenza A strains (six RCTs, VE 64%, 95% CI 23% to 82%) [11,14,15,45,47,49], and matched influenza A strains (seven RCTs, VE 61%, 95% CI 46% to 73%) [13-15,45-47,49]. The TIV also afforded protection against mismatched influenza B strains (eight RCTs, VE 52%, 95% CI 19% to 72%) [11,13-15,45-47,49], as well as matched influenza B strains (four RCTs, VE 77%, 95% CI 18% to 94%) [11,15,47,49] among adults.

### Other vaccines compared to placebo

In all, 3 RCTs including 5 influenza seasons and 1,578 participants provided efficacy estimates against mismatched strains for other vaccines [12,26,29,31]. Protection against mismatched strains was statistically significant (three RCTs, VE 56%, 95% CI 23% to 75%) [12,26,29].

Other vaccines contributed data on 5 influenza seasons from 4 RCTs including 15,592 participants for matched strains. Other vaccines provided a VE of 54% (four RCTs, 95% CI 5% to 78%) [24,29,48,51].

#### Post hoc subgroup analysis

Our results did not change after a *post hoc* sensitivity analysis was conducted to examine the influence of categorizing the vaccines as being either an inactivated influenza vaccine or a live influenza vaccine versus categorizing the vaccines as LAIV, TIV, and other.

## Discussion

In the present work, we conducted a systematic review to estimate the protection afforded by mismatched influenza vaccines. Our results show that mismatched vaccines can reduce the risk of PCR or culture-confirmed influenza by 60% for LAIV (95% CI 44% to 71%) and by 56% for TIV (95% CI 43% to 66%). These results suggest a benefit of vaccines in preventing laboratory-confirmed influenza even when there is a mismatch between vaccine composition and circulating strains. For matched influenza, point estimates for VE were slightly higher for both the LAIV (77%, 95% CI 67% to 86%) and the TIV (65%, 95% CI 57% to 72%), versus mismatched point estimates. However, there was substantial overlap in the CIs for matched and mismatched estimates in some instances; the impact of this overlap could not be formally tested via meta-analyses techniques.

A previous systematic review on a similar topic did not report the results separately for matched or mismatched strains [2]. A Cochrane review among healthy adults found a VE for TIV non-WHO matching strains (including when the specific strains are unknown) of 44% (95% CI 23% to 59%) [6]. This result was based on six influenza seasons from four RCTs. A VE of 68% (95% CI 44% to 81%) was observed for LAIV non-WHO matching strains (including when the specific strains are unknown), based on four point estimates from three RCTs [6]. Our review is based on an additional 13 RCTs and we have included more than double the data for both LAIV and TIV compared to the Cochrane review. Furthermore, here we have followed a rigorous process of identifying matched and mismatched data, which has not been attempted in previous reviews. Our results are consistent with those found in a pooled observational study including 5 years of data [53].

We found that the LAIV was more efficacious among children versus adults, which is likely a reflection of the difference in previously acquired influenza infections between age groups and the consequently larger amount of pre-vaccination antibody, which affects the live vaccine. However, we found higher point estimates for adults versus children for mismatched LAIV estimates. This finding might suggest that there might be a possible discrepancy in the degree of matching, which may have impacted our results. Specifically, trials conducted among children may have had a greater degree of mismatch than those conducted among adults. Unfortunately, the current analysis does not allow the degree of mismatch to be examined. For the purposes of our analysis, we dichotomized cross protection but in reality, the degree of mismatch is a continuum. The cross protection inferred by mismatch strains should be analyzed as a continuum in the future.

The results from our secondary outcome analysis were often inconsistent with our primary outcome results. This inconsistency is likely due to differences in sensitivity and specificity of different laboratory tests over time. Indeed, the most reliable diagnostic test for clinical practice is PCR so the results from our primary outcome should be considered the most valid [54,55].

Our results are generalizable to seasonal influenza, as most of the included studies reported on this type of influenza. Only one of the included RCTs occurred during the influenza pandemic [51]. When this study was removed via sensitivity analysis, we did not observe any differences on our meta-analysis results.

We identified three RCTs reporting data among seniors [33,36,51], yet none reported our primary outcome of interest or provided data on mismatched influenza. This finding is consistent with previous influenza reviews, for which few RCTs were identified among this age group [2,5]. Future RCTs are needed in this area to provide patients, healthcare providers, and health policymakers with guidance related to vaccination among seniors during seasons when the vaccine composition does not match circulating strains [5].

There are a few limitations in the evidence base summarized for this review. Many of the trials had an unclear risk of bias because of poor reporting. However, we did note improvements in risk of bias over time that may be because of uptake of the Consolidated Standards of Reporting Trials (CONSORT Statement), providing guidance on what should be reported in RCTs [56]. Enhanced reporting of outcome definitions might be due to clinical trial registry requirements for trials. We also noted that more trials in this area were funded by industry over time.

Another potential limitation is that although unpublished data was only obtained from six RCTs (Additional file 4), no data were obtained from unpublished RCTs. This finding suggests that we might be missing data from unpublished trials. However, we contacted many trial authors for unpublished data and searched trial registries (for example, *meta*-Register) to identify potentially relevant unpublished RCTs. Furthermore, our funnel plots did not suggest that publication bias influenced our results [23].

A critical limitation, which may have influenced our results, is the determination of mismatch between circulating strains and those found in the vaccine. Characterizing strains as antigenically similar or distinct using HI assay or ferret antisera might be insensitive, leading to misclassification of strains [57]. Residual misclassification of matching due to the limited discriminatory ability of H1 assays may have also explained some of our findings, and is a limitation of the inferences that can be made. Furthermore, the cut-off values recommended by the CDC to distinguish between match and mismatch strains using the HI assay changed during the study period across the included RCTs, although most of the studies included here used a fourfold quotient HI cut-off (Table 1). As such, some of the data from trials labeled as matched might actually have been mismatched [58], and vice versa.

## Conclusions

By summarizing the point estimates and confidence intervals for cross protection, these data can help public health officials anticipate the possible infection and complications during mismatched years. Estimates of protection for mismatched influenza seasons can be used by patients who are contemplating immunization, since the LAIV and TIV have been shown to offer benefit during matched seasons, as well as mismatched seasons.

## Competing interests

ACT, DH, MT, CTB, and ML have received consulting fees from GlaxoSmithKline. CTB has received a grant from GlaxoSmithKline. ML received payment for medical writing. St Michael's Hospital received a grant to conduct this research. ACT and ML have received financial support for travel to meetings. AC and GM are paid employees of GlaxoSmithKline and also own company stock. CS has nothing to declare. This systematic review was funded by GlaxoSmithKline, Canada. The funders had no role in study design, data collection and analysis, decision to publish, or preparation of the manuscript.

## Authors’ contributions

ACT conceived the study, designed the study, screened citations and full text articles, abstracted the data, analyzed the data, interpreted the data, and wrote the manuscript. AC conceived the study and edited the manuscript. CS screened citations and full text articles, abstracted the data, generated tables, and edited the manuscript. DH screened citations and full text articles and edited the manuscript. GM conceived the study and edited the manuscript. MHC conducted the statistical analysis, interpreted the data and edited the manuscript. MK screened citations and full text articles and edited the manuscript. CB conceived the study and edited the manuscript. ML conceived the study, designed the study, interpreted the data and edited the manuscript. All authors have read and approved the final manuscript.

## Pre-publication history

The pre-publication history for this paper can be accessed here:

http://www.biomedcentral.com/1741-7015/11/153/prepub

## Supplementary Material

Additional file 1 Study characteristics. Click here for file

Additional file 2 Other methodological concerns. Click here for file

Additional file 3 Meta-analysis results. Click here for file

Additional file 4 Unpublished data from other authors. Click here for file

## References

[B1] Fiore AE Uyeki TM Broder K Finelli L Euler GL Singleton JA Iskander JK Wortley PM Shay DK Bresee JS Cox NJ Prevention and control of influenza with vaccines: recommendations of the Advisory Committee on Immunization Practices (ACIP), 2010 MMWR Recomm Rep 2010 59 1 62 20689501

[B2] Osterholm MT Kelley NS Sommer A Belongia EA Efficacy and effectiveness of influenza vaccines: a systematic review and meta-analysis Lancet Infect Dis 2012 12 36 44 10.1016/S1473-3099(11)70295-X 22032844

[B3] Ampofo WK Baylor N Cobey S Cox NJ Daves S Edwards S Ferguson N Grohmann G Hay A Katz J Kullabutr K Lambert L Levandowski R Mishra AC Monto A Siqueira M Tashiro M Waddell AL Wairagkar N Wood J Zambon M Zhang W WHO Writing Group Improving influenza vaccine virus selection: report of a WHO informal consultation held at WHO headquarters, Geneva, Switzerland, 14–16 June 2010 Influenza Other Respi Viruses 2012 6 142 152 e141-14521819547

[B4] Centers for Disease Control and Prevention Seasonal Influenza (Flu)- Past Weekly Surveillance Reports Book Seasonal Influenza (Flu)- Past Weekly Surveillance Reports 2012 Atlanta, GA: Centers for Disease Control and Prevention

[B5] Jefferson T Di Pietrantonj C Al-Ansary LA Ferroni E Thorning S Thomas RE Vaccines for preventing influenza in the elderly Cochrane Database Syst Rev 2010 2 CD004876

[B6] Jefferson T Di Pietrantonj C Rivetti A Bawazeer GA Al-Ansary LA Ferroni E Vaccines for preventing influenza in healthy adults Cochrane Database Syst Rev 2010 7 CD001269 10.1002/14651858.CD001269.pub4 20614424

[B7] Jefferson T Rivetti A Di Pietrantonj C Demicheli V Ferroni E Vaccines for preventing influenza in healthy children Cochrane Database Syst Rev 2012 8 CD004879 10.1002/14651858.CD004879.pub4 PMC6478137 22895945

[B8] Moher D Liberati A Tetzlaff J Altman DG Preferred reporting items for systematic reviews and meta-analyses: the PRISMA statement BMJ 2009 339 b2535 10.1136/bmj.b2535 19622551 PMC2714657

[B9] Tricco AC Chit A Hallett D Soobiah C Meier G Chen M Tashkandi M Bauch C Loeb M Effect of influenza vaccines against mismatched strains: a systematic review protocol Syst Rev 2012 1 35 10.1186/2046-4053-1-35 22846340 PMC3488466

[B10] Bracco Neto H Farhat CK Tregnaghi MW Madhi SA Razmpour A Palladino G Small MG Gruber WC Forrest BD Efficacy and safety of 1 and 2 doses of live attenuated influenza vaccine in vaccine-naive children Pediatr Infect Dis J 2009 28 365 371 10.1097/INF.0b013e31819219b8 19395948

[B11] Ohmit SE Victor JC Rotthoff JR Teich ER Truscon RK Baum LL Rangarajan B Newton DW Boulton ML Monto AS Prevention of antigenically drifted influenza by inactivated and live attenuated vaccines N Engl J Med 2006 355 2513 2522 10.1056/NEJMoa061850 17167134 PMC2614682

[B12] Treanor JJ Schiff GM Hayden FG Brady RC Hay CM Meyer AL Holden-Wiltse J Liang H Gilbert A Cox M Safety and immunogenicity of a baculovirus-expressed hemagglutinin influenza vaccine: a randomized controlled trial JAMA 2007 297 1577 1582 10.1001/jama.297.14.1577 17426277

[B13] Ohmit SE Victor JC Teich ER Truscon RK Rotthoff JR Newton DW Campbell SA Boulton ML Monto AS Prevention of symptomatic seasonal influenza in 2005-2006 by inactivated and live attenuated vaccines J Infect Dis 2008 198 312 317 10.1086/589885 18522501 PMC2613648

[B14] Monto AS Ohmit SE Petrie JG Johnson E Truscon R Teich E Rotthoff J Boulton M Victor JC Comparative efficacy of inactivated and live attenuated influenza vaccines N Engl J Med 2009 361 1260 1267 10.1056/NEJMoa0808652 19776407

[B15] Jackson LA Gaglani MJ Keyserling HL Balser J Bouveret N Fries L Treanor JJ Safety, efficacy, and immunogenicity of an inactivated influenza vaccine in healthy adults: a randomized, placebo-controlled trial over two influenza seasons BMC Infect Dis 2010 10 71 10.1186/1471-2334-10-71 20236548 PMC2845585

[B16] Higgins JP Altman DG Gotzsche PC Juni P Moher D Oxman AD Savovic J Schulz KF Weeks L Sterne JA The Cochrane Collaboration’s tool for assessing risk of bias in randomised trials BMJ 2011 343 d5928 10.1136/bmj.d5928 22008217 PMC3196245

[B17] Lexchin J Bero LA Djulbegovic B Clark O Pharmaceutical industry sponsorship and research outcome and quality: systematic review BMJ 2003 326 1167 1170 10.1136/bmj.326.7400.1167 12775614 PMC156458

[B18] American College Health Association Influenza-like illness case definition http://www.acha.org/ILI_Project/ILI_case_definition_CDC.pdf

[B19] Shaw MW Xu X Li Y Normand S Ueki RT Kunimoto GY Hall H Klimov A Cox NJ Subbarao K Reappearance and global spread of variants of influenza B/Victoria/2/87 lineage viruses in the 2000–2001 and 2001–2002 seasons Virology 2002 303 1 8 10.1006/viro.2002.1719 12482653

[B20] Rota PA Wallis TR Harmon MW Rota JS Kendal AP Nerome K Cocirculation of two distinct evolutionary lineages of influenza type B virus since 1983 Virology 1990 175 59 68 10.1016/0042-6822(90)90186-U 2309452

[B21] DerSimonian R Laird N Meta-analysis in clinical trials Controlled Clin Trials 1986 7 177 188 10.1016/0197-2456(86)90046-2 3802833

[B22] Higgins JP Thompson SG Quantifying heterogeneity in a meta-analysis Stat Med 2002 21 1539 1558 10.1002/sim.1186 12111919

[B23] Egger M Davey Smith G Schneider M Minder C Bias in meta-analysis detected by a simple, graphical test BMJ 1997 315 629 634 10.1136/bmj.315.7109.629 9310563 PMC2127453

[B24] Leibovitz A Coultrip RL Kilbourne ED Legters LJ Smith CD Chin J Schulman JL Correlated studies of a recombinant influenza-virus vaccine. IV. Protection against naturally occurring influenza in military trainees J Infect Dis 1971 124 481 487 10.1093/infdis/124.5.481 5115671

[B25] Beutner KR Chow T Rubi E Strussenberg J Clement J Ogra PL Evaluation of a neuraminidase-specific influenza A virus vaccine in children: antibody responses and effects on two successive outbreaks of natural infection J Infect Dis 1979 140 844 850 10.1093/infdis/140.6.844 396336

[B26] Rytel MW Jackson LJ Niebojewski RA Haagensen JL Rosenkranz MA Field trial of live attenuated influenza A/B (“Alice”/R-75) vaccine Am J Epidemiol 1977 105 49 55 831465 10.1093/oxfordjournals.aje.a112355

[B27] Monto AS Miller FD Maassab HF Evaluation of an attenuated, cold-recombinant influenza B virus vaccine J Infect Dis 1982 145 57 64 10.1093/infdis/145.1.57 7054318

[B28] Tannock GA Bryce DA Hensley MJ Saunders NA Gillett RS Kennedy WS Responses to one or two doses of a deoxycholate subunit influenza vaccine in a primed population Vaccine 1984 2 100 106 10.1016/S0264-410X(98)90040-8 6531951

[B29] Keitel WA Cate TR Couch RB Huggins LL Hess KR Efficacy of repeated annual immunization with inactivated influenza virus vaccines over a five year period Vaccine 1997 15 1114 1122 10.1016/S0264-410X(97)00003-0 9269055

[B30] Gruber WC Taber LH Glezen WP Clover RD Abell TD Demmler RW Couch RB Live attenuated and inactivated influenza vaccine in school-age children Am J Dis Child 1990 144 595 600 2330929 10.1001/archpedi.1990.02150290089035

[B31] Edwards KM Dupont WD Westrich MK Plummer WD Jr Palmer PS Wright PF A randomized controlled trial of cold-adapted and inactivated vaccines for the prevention of influenza A disease J Infect Dis 1994 169 68 76 10.1093/infdis/169.1.68 8277200

[B32] Clover RD Crawford S Glezen WP Taber LH Matson CC Couch RB Comparison of heterotypic protection against influenza A/Taiwan/86 (H1N1) by attenuated and inactivated vaccines to A/Chile/83-like viruses J Infect Dis 1991 163 300 304 10.1093/infdis/163.2.300 1988512

[B33] Govaert TM Thijs CT Masurel N Sprenger MJ Dinant GJ Knottnerus JA The efficacy of influenza vaccination in elderly individuals. A randomized double-blind placebo-controlled trial JAMA 1994 272 1661 1665 10.1001/jama.1994.03520210045030 7966893

[B34] Powers DC Smith GE Anderson EL Kennedy DJ Hackett CS Wilkinson BE Volvovitz F Belshe RB Treanor JJ Influenza A virus vaccines containing purified recombinant H3 hemagglutinin are well tolerated and induce protective immune responses in healthy adults J Infect Dis 1995 171 1595 1599 10.1093/infdis/171.6.1595 7769297

[B35] Belshe RB Mendelman PM Treanor J King J Gruber WC Piedra P Bernstein DI Hayden FG Kotloff K Zangwill K Iacuzio D Wolff M The efficacy of live attenuated, cold-adapted, trivalent, intranasal influenzavirus vaccine in children N Engl J Med 1998 338 1405 1412 10.1056/NEJM199805143382002 9580647

[B36] Rudenko LG Arden NH Grigorieva E Naychin A Rekstin A Klimov AI Donina S Desheva J Holman RC DeGuzman A Cox NJ Katz JM Immunogenicity and efficacy of Russian live attenuated and US inactivated influenza vaccines used alone and in combination in nursing home residents Vaccine 2001 19 308 318 10930686 10.1016/s0264-410x(00)00153-5

[B37] Belshe RB Gruber WC Mendelman PM Cho I Reisinger K Block SL Wittes J Iacuzio D Piedra P Treanor J King J Kotloff K Bernstein DI Hayden FG Zangwill K Yan L Wolff M Efficacy of vaccination with live attenuated, cold-adapted, trivalent, intranasal influenza virus vaccine against a variant (A/Sydney) not contained in the vaccine J Pediatr 2000 136 168 175 10.1016/S0022-3476(00)70097-7 10657821

[B38] Bridges CB Thompson WW Meltzer MI Reeve GR Talamonti WJ Cox NJ Lilac HA Hall H Klimov A Fukuda K Effectiveness and cost-benefit of influenza vaccination of healthy working adults: a randomized controlled trial JAMA 2000 284 1655 1663 10.1001/jama.284.13.1655 11015795

[B39] Hoberman A Greenberg DP Paradise JL Rockette HE Lave JR Kearney DH Colborn DK Kurs-Lasky M Haralam MA Byers CJ Zoffel LM Fabian IA Bernard BS Kerr JD Effectiveness of inactivated influenza vaccine in preventing acute otitis media in young children: a randomized controlled trial JAMA 2003 290 1608 1616 10.1001/jama.290.12.1608 14506120

[B40] Tam JS Capeding MR Lum LC Chotpitayasunondh T Jiang Z Huang LM Lee BW Qian Y Samakoses R Lolekha S Rajamohanan KP Narayanan SN Kirubakaran C Rappaport R Razmpour A Gruber WC Forrest BD Pan-Asian CAIV-T Pediatric Efficacy Trial Network Efficacy and safety of a live attenuated, cold-adapted influenza vaccine, trivalent against culture-confirmed influenza in young children in Asia Pediatr Infect Dis J 2007 26 619 628 10.1097/INF.0b013e31806166f8 17596805

[B41] Vesikari T Fleming DM Aristegui JF Vertruyen A Ashkenazi S Rappaport R Skinner J Saville MK Gruber WC Forrest BD Safety, efficacy, and effectiveness of cold-adapted influenza vaccine-trivalent against community-acquired, culture-confirmed influenza in young children attending day care Pediatrics 2006 118 2298 2312 10.1542/peds.2006-0725 17142512

[B42] Forrest BD Pride MW Dunning AJ Capeding MR Chotpitayasunondh T Tam JS Rappaport R Eldridge JH Gruber WC Correlation of cellular immune responses with protection against culture-confirmed influenza virus in young children Clin Vaccine Immunol 2008 15 1042 1053 10.1128/CVI.00397-07 18448618 PMC2446637

[B43] Lum LC Borja-Tabora CF Breiman RF Vesikari T Sablan BP Chay OM Tantracheewathorn T Schmitt HJ Lau YL Bowonkiratikachorn P Tam JS Lee BW Tan KK Pejcz J Cha S Gutierrez-Brito M Kaltenis P Vertruyen A Czajka H Bojarskas J Brooks WA Cheng SM Rappaport R Baker S Gruber WC Forrest BD Influenza vaccine concurrently administered with a combination measles, mumps, and rubella vaccine to young children Vaccine 2010 28 1566 1574 10.1016/j.vaccine.2009.11.054 20003918

[B44] Langley JM Aoki F Ward BJ McGeer A Angel JB Stiver G Gorfinkel I Shu D White L Lasko B Dzongowski P Papp K Alexander M Boivin G Fries L A nasally administered trivalent inactivated influenza vaccine is well tolerated, stimulates both mucosal and systemic immunity, and potentially protects against influenza illness Vaccine 2011 29 1921 1928 10.1016/j.vaccine.2010.12.100 21219987

[B45] Beran J Wertzova V Honegr K Kaliskova E Havlickova M Havlik J Jirincova H Van Belle P Jain V Innis B Devaster JM Challenge of conducting a placebo-controlled randomized efficacy study for influenza vaccine in a season with low attack rate and a mismatched vaccine B strain: a concrete example BMC Infect Dis 2009 9 2 10.1186/1471-2334-9-2 19149900 PMC2639595

[B46] Beran J Vesikari T Wertzova V Karvonen A Honegr K Lindblad N Van Belle P Peeters M Innis BL Devaster JM Efficacy of inactivated split-virus influenza vaccine against culture-confirmed influenza in healthy adults: a prospective, randomized, placebo-controlled trial J Infect Dis 2009 200 1861 1869 10.1086/648406 19909082

[B47] Frey S Vesikari T Szymczakiewicz-Multanowska A Lattanzi M Izu A Groth N Holmes S Clinical efficacy of cell culture-derived and egg-derived inactivated subunit influenza vaccines in healthy adults Clinical Infect Dis 2010 51 997 1004 10.1086/656578 20868284

[B48] Treanor JJ El Sahly H King J Graham I Izikson R Kohberger R Patriarca P Cox M Protective efficacy of a trivalent recombinant hemagglutinin protein vaccine (FluBlok(R)) against influenza in healthy adults: a randomized, placebo-controlled trial Vaccine 2011 29 7733 7739 10.1016/j.vaccine.2011.07.128 21835220

[B49] Barrett PN Berezuk G Fritsch S Aichinger G Hart MK El-Amin W Kistner O Ehrlich HJ Efficacy, safety, and immunogenicity of a Vero-cell-culture-derived trivalent influenza vaccine: a multicentre, double-blind, randomised, placebo-controlled trial Lancet 2011 377 751 759 10.1016/S0140-6736(10)62228-3 21329971

[B50] Cowling BJ Ng S Ma ES Cheng CK Wai W Fang VJ Chan KH Ip DK Chiu SS Peiris JS Leung GM Protective efficacy of seasonal influenza vaccination against seasonal and pandemic influenza virus infection during 2009 in Hong Kong Clinical Infect Dis 2010 51 1370 1379 10.1086/657311 21067351

[B51] Talaat KR Greenberg ME Lai MH Hartel GF Wichems CH Rockman S Jeanfreau RJ Ghosh MR Kabongo ML Gittleson C Karron RA A single dose of unadjuvanted novel 2009 H1N1 vaccine is immunogenic and well tolerated in young and elderly adults J Infect Dis 2010 202 1327 1337 10.1086/656601 20874515

[B52] Keitel WA Cate TR Couch RB Efficacy of sequential annual vaccination with inactivated influenza virus vaccine Am J Epidemiol 1988 127 353 364 3337087 10.1093/oxfordjournals.aje.a114809

[B53] Kelly HA Sullivan SG Grant KA Fielding JE Moderate influenza vaccine effectiveness with variable effectiveness by match between circulating and vaccine strains in Australian adults aged 20–64 years, 2007–2011 Influenza Other Respi Virusesin press 10.1111/irv.12018 PMC5781205 23078073

[B54] Landry ML Diagnostic tests for influenza infection Curr Opin Pediatr 2011 23 91 97 10.1097/MOP.0b013e328341ebd9 21150446

[B55] Petrie JG Ohmit SE Johnson E Cross RT Monto AS Efficacy studies of influenza vaccines: effect of end points used and characteristics of vaccine failures J Infect Dis 2011 203 1309 1315 10.1093/infdis/jir015 21378375 PMC3069734

[B56] Schulz KF Altman DG Moher D CONSORT 2010 Statement: Updated guidelines for reporting parallel group randomised trials J Clin Epidemiol 2010 63 834 840 10.1016/j.jclinepi.2010.02.005 20346629

[B57] Smith DJ Lapedes AS de Jong JC Bestebroer TM Rimmelzwaan GF Osterhaus AD Fouchier RA Mapping the antigenic and genetic evolution of influenza virus Science 2004 305 371 376 10.1126/science.1097211 15218094

[B58] Skowronski DM Janjua NZ De Serres G Winter AL Dickinson JA Gardy JL Gubbay J Fonseca K Charest H Crowcroft NS Fradet MD Bastien N Li Y Krajden M Sabaiduc S Petric M A sentinel platform to evaluate influenza vaccine effectiveness and new variant circulation, Canada 2010–2011 season Clinical Infect Dis 2012 55 332 342 10.1093/cid/cis431 22539661

[B59] Belshe RB Coelingh K Ambrose CS Woo JC Wu X Efficacy of live attenuated influenza vaccine in children against influenza B viruses by lineage and antigenic similarity Vaccine 2012 28 2149 2156 20003926 10.1016/j.vaccine.2009.11.068

