# Supplementary material for: Comparing influenza vaccine efficacy against mismatched and matched strains: a systematic review and meta-analysis
Source: BMC Med. 2013 Jun 25;11:153. doi: 10.1186/1741-7015-11-153 (PMC3706345; doi:10.1186/1741-7015-11-153)
Supplement: Additional file 1 — Study characteristics. [file 1741-7015-11-153-S1.doc]

**Additional file 1**. Study Characteristics

| **Author (YR)** | **Country of Conduct / YR** | **Follow-up time (mos)** | **Vaccine Type** | **Placebo composition** | **Route of administration** | **Concentration of vaccine** | **Dose** |
| --- | --- | --- | --- | --- | --- | --- | --- |
| Leibovitz (1971) | USA / 1970 | 1.8 | Other (MIV) | Did not receive anything | SC or IM | 556 CCA units/mL | 1.0 mL |
| Beutner (1979) | USA / 1974 | 7 | Other (MIV) | Vaccine dilutent | IM | NR | 0.5mL |
| Rytel (1977) | USA/1974 | 6 | Other (Bivalent attenuated vaccine) | NR | IN | 10^7.2-7.8^ EID_50_ | NR |
| Monto (1982) | USA/1979 | 6 | Other (Bivalent attenuated vaccine) | Hanks balanced salt solution | IN | 10^5.8^ EID_50_ | 0.5mL |
| Tannock (1984) | Australia/ 1981 | 6 | TIV | Sterile saline solution | SC | 7µg | NR |
| Keitel (1997) § ; Keitel (1988) | USA/1983-1988 | Post influenza season | Other (WV) | Saline | IM | Y1-2:15 µg  Y3-5: NR | Y1-3,5: 0.5mL, Y4: 1.0mL |
| Gruber (1990) | USA/1985 | Post influenza season | TIV | Saline | IM | 15µg | 0.5mL |
| Edwards (1994) | USA/1986-1990 | Post influenza season | LAIV, TIV | Allatonic fluid | IN, IM | **LAIV Y1-2,4:**  10^7.0-7.6^pfu/ml, **Y3:** 0^6.0^pfu/mL  **TIV:** 15µg | 0.5mL-1.0mL (depending on age group) |
| Clover (1991) | USA /1989 | Post influenza season | TIV, Other (Bivalent CR) | **TIV:** sterile saline  **Bivalent CR:** Buffered saline | IM, IN | **TIV:** 15 µg  **Bivalent CR**: 10^7.0^ TCID_50_ | **TIV/ Bivalent CR:**0.5mL |
| Govaert (1994) | Netherlands/1991 | 5 | Other (purified split viron vaccine) | Saline | IM | 15µg | 0.5mL |
| Powers (1995) | USA/1993 | 6 | TIV | Saline | IM | 15µg | 0.5mL |
| Belshe (1998) | USA/1996 | 4 | LAIV | egg allantoic fluid containing sucrose, phosphate, and glutamate | IN | 10^6.7^TCID_50_ | 0.5mL |
| Rudenko (2001) | Russia/1996 | Post influenza season | LAIV, TIV | **LAIV:**  lyophilized allantoic fluid of uninfected embryonated chicken egg **TIV:**Saline | IM, IN | **LAIV:**10^6.5-7.2^ EID_50_  **TIV:** 15µg, | **LAIV:**  1.0 mL  **TIV:** 0.5mL |
| Belshe (2000) | USA/ 1997 | 4 | LAIV | egg allantoic fluid containing sucrose-phosphate-glutamate | IN | 10^7.0^TCID | 0.5mL |
| Bridges (2000) | USA/1997-1999 | 12 | TIV | Sterile saline | IM | NR | NR |
| Hoberman (2003) | USA/1999-2001 | 12 | TIV | Standard dilutent | IM | NR | 0.25mL |
| Tam (2007) | Multi-site trial in Asia/2000-2002 | 8-12 | LAIV | Sterile saline | IN | 10^7.0^TCID | 0.2mL |
| Vesikari (2006) | Multi-site trial in Europe and Israel/2000-2002 | Post influenza season | LAIV | Sterile saline solution | IN | 10^7.0^TCID | 0.2mL |
| Forrest (2008) | Multi-site in Asia/2001 | 9 | LAIV | Sterile saline | IN | 10^7.0±0.5^ FFU/mL | 0.2mL |
| BraccoNeto (2009) * | Multi-site trial in South Africa and South America/ 2001-2003 | Post influenza season | LAIV | Saline | IN | 10^7.0±0.5^FFU/ml | 0.2mL |
| Lum (2010) | Multi-site trial in Asia, Europe and South America /2002 | Post influenza season | LAIV | NR | IN | 10^7.0^ TCID | 0.2mL |
| Langley (2011) | Canada/ 2003 | 6.5 | TIV | Sterile phospate-buffered isotonic saline | IN | 15μg | 0.2mL |
| Ohmit (2006) * | USA/2004 | 5 | LAIV, TIV | Saline | IN, IM | **LAIV**: 10^6.5-7.5^FFU/mL  **TIV**: 15μg, | **LAIV/TIV**:0.5mL |
| Treanor (2007) * | USA/2004 | 6.4 | Other (purified rHAO vaccine) | Saline | IM | 135μg | 0.5mL |
| Beran (2009a) | Czech Republic/ 2005 | Post influenza season | TIV | Saline | IM | 15µg | 0.5mL |
| Jackson (2010) | USA/2005 | 3.7 | TIV | Saline | IM | 15µg | 0.5mL |
| Ohmit (2008) * | USA/2005 | Post influenza season | LAIV, TIV | Physiological saline | IN, IM | **LAIV:** 10^6.5-7.5^TCID_50_  **TIV:** 15μg | 0.5 mL |
| Beran (2009b) | Multi-site trial Europe/ 2006 | Post influenza season | TIV | Saline | IM | 15μg | 0.5 mL |
| Monto (2009)* | USA/2007 | 5 | LAIV, TIV | Physiological saline | IM, IN | **LAIV:**  10^6.5-7.5^FFU/mL  **TIV:**15μg | **LAIV:** 0.2mL  **TIV:** 0.5mL |
| Frey (2010) | Multi-site trial North America and Europe/ 2007 | 9 | LAIV, TIV | Phosphate buffered saline | IM | 15μg | 0.5mL |
| Treanor (2011) | USA/2007 | Post influenza season | Other (FluBlok®) | Saline | IM | 45μg | NR |
| Barrett (2011) | Multi-site trial in USA/  2008 | 6 | TIV | Phosphate buffered saline | IM | 15µg | 0.5mL |
| Cowling (2010) | Hong Kong/  2008 | 11 | TIV | Saline | IM | NR | 0.5mL |
| Talaat (2010) | USA/  2009 | Post influenza season | Other (MIV) | vaccine dilutent and thimerosal | IM | 15 µg | 0.5mL |

**Note:** * unpublished data was obtained from the author § main publication **Abbreviations**: CCA chick-cell-agglutinating, EID egg infectious dose, FFU phosphorescent focus units, IM intramuscular, IN intranasal spray, LAIV live attenuated vaccine, MIV Monovalent inactivated vaccine, mL milliliters, NR not reported, pfu plaque forming units, RT-PCR reverse transcriptase polymerase chain, SC subcutaneous injection, TCID tissue concentration infectious dose, TIV trivalent inactivated vaccine, μg micrograms, WV whole viral vaccine, USA Unites States of America, YR year.
