# Supplementary material for: Comparing influenza vaccine efficacy against mismatched and matched strains: a systematic review and meta-analysis
Source: BMC Med. 2013 Jun 25;11:153. doi: 10.1186/1741-7015-11-153 (PMC3706345; doi:10.1186/1741-7015-11-153)
Supplement: Additional file 2 — Other methodological concerns. [file 1741-7015-11-153-S2.doc]

**Additional file 2. Other Methodological Concerns**

| **Author (YR)** | **Country of Conduct / YR** | **Case definition used by nurses to identify ILI**^1^ | **Surveillance system for ILI**^2^ | **Multi-site trials**^3^ |
| --- | --- | --- | --- | --- |
| Leibovitz (1971) | USA / 1970 | No | Unclear | N/A |
| Beutner (1979) | USA / 1974 | Unclear | Yes | N/A |
| Rytel (1977) | USA/1974 | No | Yes | N/A |
| Monto (1982) | USA/1979 | Unclear | Yes | N/A |
| Tannock (1984) | Australia/ 1981 | Unclear | Yes | Unclear |
| Keitel (1997) | USA/1983-1988 | Unclear | Yes | N/A |
| Gruber (1990) | USA/1985 | Unclear | Yes | Yes |
| Edwards (1994) | USA/1986-1990 | No | Yes | Yes |
| Clover (1991) | USA /1989 | Unclear | Yes | Unclear |
| Govaert (1994) | Netherlands/ 1991 | No | Yes | Unclear |
| Powers (1995) | USA/1993 | No | Yes | N/A |
| Belshe (1998) | USA/1996 | Unclear | Yes | Yes |
| Rudenko (2001) | Russia/1996 | No | Yes | Yes |
| Belshe (2000) | USA/1997 | Unclear | Yes | Unclear |
| Bridges (2000) | USA/1997-1998 | Yes | Yes | N/A |
| Hoberman (2003) | USA/1999-2000 | No | Yes | N/A |
| Tam (2007) | Multi-site trial in Asia/2000-2001 | Unclear | Yes | Unclear |
| Vesikari (2006) | Multi-site trial in Europe and Israel/2000-2001 | Unclear | Yes | Unclear |
| Bracco Neto (2009) * | Multi-site trial in South Africa and South America/ 2001-2002 | No | Yes | Unclear |
| Lum (2010) | Multi-site trial in Asia, Europe and South America /2002 | No | Yes | Unclear |
| Forrest (2008) | Multi-site in Asia/2002 | No | Yes | Unclear |
| Langley (2011) | Canada/2003 | Yes | Yes | Unclear |
| Ohmit (2006) * | USA/2004 | No | Yes | Unclear |
| Treanor (2007) * | USA/2004 | Yes | Yes | Yes |
| Beran (2009a) | Czech Republic/ 2005 | Yes | Yes | Unclear |
| Jackson (2010) | USA/2005 | No | Yes | Yes |
| Ohmit (2008) * | USA/2005 | No | Yes | Unclear |
| Beran (2009b) | Multi-site trial Europe/ 2006 | No | Yes | Unclear |
| Monto (2009)* | USA/2007 | No | Yes | Unclear |
| Frey (2010) | Multi-site trial North America and Europe/ 2007 | Yes | Yes | Unclear |
| Treanor (2011) | USA/2007 | Yes | Yes | Yes |
| Barrett (2011) | Multi-site trial in USA/2008 | No | Yes | Unclear |
| Cowling (2010) | Hong Kong/2008 | Yes | Yes | N/A |
| Talaat (2010) | USA/2009 | No | Yes | Unclear |

**Note:** * unpublished data was obtained from the author.

**Abbreviations**: ILI influenza like illness, N/A not applicable, USA Unites States of America.

**Legend**

1. Case definition used by nurses to identify ILI was defined as fever ≥ 100° F and cough and/or sore throat (in the absence of a known cause other than influenza). A “yes” response means they study used the same definition as above, “no” means study provided a definition not matching the above definition, “unclear” means a definition was not provided
2. Surveillance system for ILI was defined as a system in place to track influenza-like-illness in the community or study population. A “yes” response means a nurses contacted participants to see if they had cough, fever, sore throat etc. A “no” response means no follow-up and, an “unclear” means the paper did not specify or were not clear on type of follow-up.
3. A multi-site trial refers to the randomization of participants across multiple sites. A “yes” means randomization was balanced at each site, meaning the overall vaccine: placebo ratio is the same across all sites. A “no” response means randomization was unbalanced or vaccine: placebo ratio is not consistent across all sites. An “unclear” response means randomization by site was not specified.
