# Supplementary material for: Comparing influenza vaccine efficacy against mismatched and matched strains: a systematic review and meta-analysis
Source: BMC Med. 2013 Jun 25;11:153. doi: 10.1186/1741-7015-11-153 (PMC3706345; doi:10.1186/1741-7015-11-153)
Supplement: Additional file 3 — Meta-analysis results. [file 1741-7015-11-153-S3.doc]

**Additional file 3**. Meta-analysis results

| **Age category** | **Type of influenza** | **# Influenza seasons** | **# Patients** | **# Influenza cases (vaccine group)** | **# Influenza cases (control group)** | **I^2^** | **VE (95% CI)** |
| --- | --- | --- | --- | --- | --- | --- | --- |
| **LAIV MISMATCHED – Primary Analysis** | | | | | | | |
| All studies | All influenza | 15 | 27098 | 216 | 416 | 0.67 | 0.60 (0.44, 0.71)* |
| All studies | Influenza A | 10 | 22099 | 63 | 188 | 0.49 | 0.76 (0.61, 0.85)* |
| All studies | Influenza B | 12 | 21897 | 154 | 232 | 0.08 | 0.44 (0.31, 0.55)* |
| All studies | B lineage | 12 | 21897 | 115 | 169 | 0 | 0.43 (0.16, 0.66)* |
| All studies | B drift | 3 | 5377 | 39 | 63 | 0.10 | 0.62 (0.21, 0.81)* |
| Adults | All influenza | 6 | 10673 | 41 | 90 | 0 | 0.62 (0.44, 0.73)* |
| Adults | Influenza A | 5 | 9482 | 5 | 23 | 0.02 | 0.83 (0.52, 0.94)* |
| Adults | Influenza B | 4 | 10673 | 36 | 68 | 0 | 0.53 (0.29, 0.69)* |
| Children (6-36 mos) | All influenza | 9 | 12582 | 157 | 269 | 0.78 | 0.54 (0.28, 0.71)* |
| Children (6-36 mos) | Influenza A | 5 | 8774 | 40 | 108 | 0.67 | 0.75 (0.41, 0.90)* |
| Children (6-36 mos) | Influenza B | 8 | 11224 | 118 | 164 | 0.25 | 0.42 (0.22, 0.56)* |
| Children (6-36 mos) | B lineage | 8 | 11224 | 79 | 101 | 0.10 | 0.34 (-0.04, 0.59) |
| Children (6-36 mos) | B drift | 3 | 5377 | 39 | 63 | 0.10 | 0.62 (0.21, 0.81)* |
| **LAIV MISMATCHED – Secondary Analysis** | | | | | | | |
| All studies | All influenza | 3 | 4157 | 165 | 48 | 0.77 | -0.94 (-7.14, 0.54) |
| All studies | Influenza A | 2 | 3843 | 163 | 242 | 0 | 0.32 (-0.14, 0.59) |
| All studies | Influenza B | 3 | 4127 | 3 | 250 | 0.81 | 0.98 (0.87, 1.00)* |
| **LAIV MATCHED – Primary Analysis** | | | | | | | |
| All studies | All influenza | 15 | 28114 | 255 | 844 | 0.85 | 0.77 (0.66,0.85)* |
| All studies | Influenza A | 14 | 27389 | 222 | 732 | 0.86 | 0.79 (0.68,0.87)* |
| All studies | Influenza B | 7 | 14752 | 35 | 135 | 0.56 | 0.77 (0.55, 0.88)* |
| Adults | All influenza | 6 | 10673 | 79 | 81 | 0.76 | 0.48 (-0.27,0.79) |
| Adults | Influenza A | 5 | 9948 | 76 | 78 | 0.86 | 0.49 (-0.52, 0.83) |
| Adults | Influenza B | 2 | 8344 | 3 | 3 | 0 | 0.44 (-1.56,0.88) |
| Children (6-36 mos) | All influenza | 9 | 13187 | 165 | 713 | 0.79 | 0.83 (0.75, 0.88)* |
| Children (6-36 mos) | Influenza A | 9 | 13187 | 135 | 604 | 0.79 | 0.84 (0.76, 0.90)* |
| Children (6-36 mos) | Influenza B | 6 | 6408 | 32 | 132 | 0.65 | 0.79 (0.58, 0.90)* |
| **LAIV MATCHED – Secondary Analysis** | | | | | | | |
| All studies | All influenza | 1 | 4254 | 119 | 265 | 0.85 | 0.54 (0.20, 0.73)* |
| **TIV MISMATCHED – Primary Analysis** | | | | | | | |
| All studies | All influenza | 13 | 44026 | 111 | 213 | 0.35 | 0.56 (0.43, 0.66)* |
| All studies | Influenza A | 9 | 35726 | 35 | 97 | 0 | 0.59 (-0.33, 0.92) |
| All studies | Influenza B | 9 | 39129 | 72 | 108 | 0 | 0.46 (0.27, 0.60)* |
| Adults | All influenza | 10 | 40027 | 95 | 155 | 0.59 | 0.52 (0.37, 0.63)* |
| Adults | Influenza A | 7 | 31721 | 19 | 39 | 0.21 | 0.64 (0.23, 0.82)* |
| Adults | Influenza B | 9 | 39129 | 72 | 108 | 0 | 0.52 (0.19, 0.72)* |
| **TIV MISMATCHED – Secondary Analysis** | | | | | | | |
| All studies | All influenza | 4 | 661 | 50 | 93 | 0.73 | 0.42 (-0.15, 0.71) |
| **TIV MATCHED – Primary Analysis** | | | | | | | |
| All studies | All influenza | 14 | 44317 | 174 | 345 | 0.12 | 0.65 (0.58, 0.72)* |
| All studies | Influenza A | 11 | 42803 | 148 | 306 | 0.11 | 0.63 (0.51, 0.72)* |
| All studies | Influenza B | 5 | 19618 | 2 | 13 | 0 | 0.77 (0.25, 0.93) * |
| Adults | All influenza | 9 | 39129 | 136 | 266 | 0.25 | 0.65 (0.54, 0.73)* |
| Adults | Influenza A | 8 | 38401 | 134 | 252 | 0.49 | 0.61 (0.46, 0.73)* |
| Adults | Influenza B | 4 | 19499 | 2 | 12 | 0 | 0.77 (0.18,0.94)* |
| Children | All influenza | 3 | 905 | 26 | 29 | 0.32 | 0.45 (-0.66, 0.92) |
| **TIV MATCHED – Secondary Analysis** | | | | | | | |
| All studies | All influenza | 6 | 2526 | 33 | 86 | 0 | 0.63 (0.44, 0.74)* |
| All studies | Influenza A | 3 | 208 | 9 | 20 | 0 | 0.65 (0.28, 0.83)* |
| **OTHER VACCINES MISMATCHED† - Primary Analysis** | | | | | | | |
| All studies | All influenza | 5 | 1578 | 20 | 60 | 0.16 | 0.56 (0.23,0.75)* |
| **OTHER VACCINES MISMATCHED† – Secondary Analysis** | | | | | | | |
| All studies | All influenza | 2 | 422 | 14 | 44 | 0 | 0.55 (0.24, 0.73)* |
| **OTHER VACCINES MATCHED† – Primary Analysis** | | | | | | | |
| All studies | All influenza | 5 | 15592 | 18 | 125 | 0.37 | 0.54 (0.05,0.78)* |
| **OTHER VACCINES MATCHED† – Secondary Analysis** | | | | | | | |
| All studies | All influenza | 3 | 2868 | 54 | 163 | 0.13 | 0.69 (0.56, 0.78)* |

**Note:** *statistically significant, † influenza B not estimated because of 0 events reported in both arms across all randomized trials.

**Abbreviations:** CI confidence interval, LAIV live attenuated influenza vaccine, RR relative risk, TIV trivalent inactivated vaccine, VE vaccine efficacy.
