# Supplementary material for: Comparing influenza vaccine efficacy against mismatched and matched strains: a systematic review and meta-analysis
Source: BMC Med. 2013 Jun 25;11:153. doi: 10.1186/1741-7015-11-153 (PMC3706345; doi:10.1186/1741-7015-11-153)
Supplement: Additional file 4 — Unpublished data from other authors. [file 1741-7015-11-153-S4.doc]

**Additional file 4. Unpublished data from authors**

**Bracco Neto Year 1**

| **Table 1: Number and Percent of Children 6 to <36 Months of Age with Culture-Confirmed Influenza Illness in the First Season of Study D153-P504 (Per Protocol Efficacy Population or as Indicated)** | | | | | | |
| --- | --- | --- | --- | --- | --- | --- |
|  | **Randomized Treatment Group^a^** | | | | | |
|  | **CC/C** | | **CS/C** | | **Placebo** | |
|  | **N^b^** | **n^c^ (%)** | **N^b^** | **n^c^ (%)** | **N^b^** | **n^c^ (%)** |
| Community-acquired subtypes antigenically similar to those in the vaccine^d^ |  |  |  |  |  |  |
| Any strain^e^ | 944 | 50 ( 5.3) | 935 | 79 ( 8.4) | 942 | 188 (20.0) |
| A/H1 | 944 | 1 ( 0.1) | 935 | 0 ( 0.0) | 942 | 0 ( 0.0) |
| A/H3 | 944 | 38 ( 4.0) | 935 | 57 ( 6.1) | 942 | 139 (14.8) |
| B | 944 | 11 ( 1.2) | 935 | 22 ( 2.4) | 942 | 59 ( 6.3) |
| Any strain: Intent-to-treat population | 1064 | 54 ( 5.1) | 1067 | 90 ( 8.4) | 1069 | 207 (19.4) |
|  | | | | | | |
| Any community-acquired subtypes |  |  |  |  |  |  |
| Any strain | 944 | 53 ( 5.6) | 935 | 82 ( 8.8) | 942 | 189 (20.1) |
| A/H1 | 944 | 1 ( 0.1) | 935 | 0 ( 0.0) | 942 | 0 ( 0.0) |
| A/H3 | 944 | 39 ( 4.1) | 935 | 59 ( 6.3) | 942 | 139 (14.8) |
| B | 944 | 13 ( 1.4) | 935 | 24 ( 2.6) | 942 | 61 ( 6.5) |
| Any strain: Intent-to-treat population | 1064 | 57 ( 5.4) | 1067 | 93 ( 8.7) | 1069 | 210 (19.6) |
| a: Treatment abbreviations: C=CAIV-T, S=S-Placebo, E=E-Placebo. The coding used to identify the randomization groups is as follows: CC/C=2 doses of C in year 1 and 1 dose of C in the second year (study group 1). CS/C=1 dose of C, then 1 dose of S in year 1 and 1 dose of C in the second year (study group 2). Placebo is the combination of the 2 groups which receive only S or E in both years (study groups 3 and 4). | | | | | | |
| b: Number of subjects in the population. | | | | | | |
| c: Number of subjects with culture-confirmed influenza illness of the indicated type/subtype. | | | | | | |
| d: The following strains isolated in this study were considered antigenically similar to those in the vaccine: A/NEWCALEDONIA/20/99-LIKE(H1), A/PANAMA/2007/99-LIKE(H3), B/YAMANASHI/166/98-LIKE, and B/VICTORIA/504/00-LIKE. | | | | | | |
| e: Primary endpoint of the study. | | | | | | |
|  | | | | | | |

**Bracco Neto Year 2**

| **Table 2: Number and Percent of Children 6 to <36 Months of Age with Culture-Confirmed Influenza Illness in the Second Season of Study D153-P504 (Per Protocol Efficacy Population or as Indicated)** | | | | | | |
| --- | --- | --- | --- | --- | --- | --- |
|  | **Randomized Treatment Group^a^** | | | | | |
|  | **CC/C** | | **CS/C** | | **Placebo** | |
|  | **N^b^** | **n^c^ (%)** | **N^b^** | **n^c^ (%)** | **N^b^** | **n^c^ (%)** |
| Community-acquired subtypes antigenically similar to those in the vaccine^d^ |  |  |  |  |  |  |
| Any strain | 338 | 6 ( 1.8) | 684 | 16 ( 2.3) | 342 | 23 ( 6.7) |
| A/H1 | 338 | 1 ( 0.3) | 684 | 9 ( 1.3) | 342 | 17 ( 5.0) |
| A/H3 | 338 | 2 ( 0.6) | 684 | 1 ( 0.1) | 342 | 4 ( 1.2) |
| B | 338 | 4 ( 1.2) | 684 | 6 ( 0.9) | 342 | 2 ( 0.6) |
| Any strain: Intent-to-treat population | 735 | 17 ( 2.3) | 732 | 18 ( 2.5) | 735 | 35 ( 4.8) |
|  | | | | | | |
| Any community-acquired subtypes |  |  |  |  |  |  |
| Any strain | 338 | 29 ( 8.6) | 684 | 59 ( 8.6) | 342 | 55 (16.1) |
| A/H1 | 338 | 1 ( 0.3) | 684 | 10 ( 1.5) | 342 | 17 ( 5.0) |
| A/H3 | 338 | 2 ( 0.6) | 684 | 1 ( 0.1) | 342 | 4 ( 1.2) |
| B | 338 | 27 ( 8.0) | 684 | 48 ( 7.0) | 342 | 36 (10.5) |
| Any strain: Intent-to-treat population | 735 | 69 ( 9.4) | 732 | 64 ( 8.7) | 735 | 83 (11.3) |
| a: Treatment abbreviations: C=CAIV-T, S=S-Placebo, E=E-Placebo. The coding used to identify the randomization groups is as follows: CC/C=2 doses of C in year 1 and 1 dose of C in the second year (study group 1). CS/C=1 dose of C, then 1 dose of S in year 1 and 1 dose of C in the second year (study group 2). Placebo is the combination of the 2 groups which receive only S or E in both years (study groups 3 and 4). | | | | | | |
| b: Number of subjects in the population. | | | | | | |
| c: Number of subjects with culture-confirmed influenza illness of the indicated type/subtype. | | | | | | |
| d: The following strains isolated in this study were considered antigenically similar to those in the vaccine: A/NEWCALEDONIA/20/99-LIKE(H1), A/PANAMA/2007/99-LIKE(H3), and B/VICTORIA/504/00-LIKE. | | | | | | |

**Ohmit 2006**

|  | **LAIV** | | **TIV** | | **Placebo** | |
| --- | --- | --- | --- | --- | --- | --- |
|  | **# influenza cases** | **Total # in group** | **# influenza cases** | **Total # in group** | **# influenza cases** | **Total # in group** |
| Mismatch (influenza A) | 4 | 519 | 4 | 522 | 6 | 206 |
| Match (influenza A) | 0 | 519 | 0 | 522 | 0 | 206 |
| Mismatch (influenza B) | 6 | 519 | 1 | 522 | 4 | 206 |
| Match (influenza B) | 3 | 519 | 2 | 522 | 2 | 206 |

**Ohmit, 2008**

|  | **LAIV** | | **TIV** | | **Placebo** | |
| --- | --- | --- | --- | --- | --- | --- |
|  | **# influenza cases** | **Total # in group** | **# influenza cases** | **Total # in group** | **# influenza cases** | **Total # in group** |
| Mismatch (influenza A) | 0 | 853 | 0 | 867 | 0 | 338 |
| Match (influenza A) | 14 | 853 | 12 | 867 | 5 | 338 |
| Mismatch (influenza B) | 0 | 853 | 0 | 867 | 1 | 338 |
| Match (influenza B) | 0 | 853 | 0 | 867 | 0 | 338 |

**Monto, 2009**

|  | **LAIV** | | **TIV** | | **Placebo** | |
| --- | --- | --- | --- | --- | --- | --- |
|  | **# influenza cases** | **Total # in group** | **# influenza cases** | **Total # in group** | **# influenza cases** | **Total # in group** |
| Mismatch (influenza A) | 0 | 813 | 0 | 814 | 1 | 325 |
| Match (influenza A) | 55 | 813 | 22 | 814 | 30 | 325 |
| Mismatch (influenza B) | 1 | 813 | 6 | 814 | 4 | 325 |
| Match (influenza B) | 0 | 813 | 0 | 814 | 0 | 325 |

**Jackson 2010 Y1**

|  | **TIV** | | **Placebo** | |
| --- | --- | --- | --- | --- |
|  | **# influenza cases** | **Total # in group** | **# influenza cases** | **Total # in group** |
| Matched A | 14 | 1702 | 30 | 1725 |
| Matched B | 0 | 1702 | 0 | 1725 |
| **Total Matched A&B** | **14** | **1702** | **30** | **1725** |
| Mismatched A | 1 | 1702 | 4 | 1725 |
| Mismatched B | 4 | 1702 | 4 | 1725 |
| **Total mismatched A& B** | **5** | **1702** | **8** | **1725** |

**Jackson 2010 Y2**

|  | **TIV** | | **Placebo** | |
| --- | --- | --- | --- | --- |
|  | **# influenza cases** | **Total # in group** | **# influenza cases** | **Total # in group** |
| Matched A | 9 | 2011 | 10 | 2043 |
| Matched B | 0 | 2011 | 5 | 2043 |
| **Total matched A&B** | **9** | **2011** | **15** | **2043** |
| Mismatched A | 1 | 2011 | 4 | 2043 |
| Mismatched B | 1 | 2011 | 3 | 2043 |
| **Total mismatched A&B** | **2** | **2011** | **7** | **2043** |

**Treanor (2007)**

|  | **rHAO Vaccine** | | **Placebo** | |
| --- | --- | --- | --- | --- |
|  | **# of influenza cases** | **Total # in group** | **# of influenza cases** | **Total # in group** |
| Matched A | 0 | 151 | 0 | 153 |
| Matched B | 0 | 151 | 0 | 153 |
| Total A & B | 0 | 151 | 0 | 153 |
| Mismatched A | 0 | 151 | 6 | 153 |
| Mismatched B* | 1 | 151 | 2 | 153 |
| Mismatched A & B | 1 | 151 | 8 | 153 |

*influenza B is a lineage variant according to author
